# Study Models of Drug–Drug Interactions Involving P-Glycoprotein: The Potential Benefit of P-Glycoprotein Modulation at the Kidney and Intestinal Levels

**DOI:** 10.3390/molecules28227532

**Published:** 2023-11-10

**Authors:** Jéssica Veiga-Matos, Ana I. Morales, Marta Prieto, Fernando Remião, Renata Silva

**Affiliations:** 1 UCIBIO-Applied Molecular Biosciences Unit, Laboratory of Toxicology, Department of Biological Sciences, Faculty of Pharmacy, University of Porto, 4050-313 Porto, Portugal; jessicamatos1996@outlook.com; 2 Associate Laboratory i4HB—Institute for Health and Bioeconomy, Faculty of Pharmacy, University of Porto, 4050-313 Porto, Portugal; 3 Toxicology Unit (Universidad de Salamanca), Group of Translational Research on Renal and Cardiovascular Diseases (TRECARD), Institute of Biomedical Research of Salamanca (IBSAL), 37007 Salamanca, Spain; amorales@usal.es (A.I.M.); martapv@usal.es (M.P.)

**Keywords:** P-glycoprotein, inhibition, induction, activation, kidney, *in silico*, *in vitro*, *ex vivo*, *in vivo*

## Abstract

P-glycoprotein (P-gp) is a crucial membrane transporter situated on the cell’s apical surface, being responsible for eliminating xenobiotics and endobiotics. P-gp modulators are compounds that can directly or indirectly affect this protein, leading to changes in its expression and function. These modulators can act as inhibitors, inducers, or activators, potentially causing drug–drug interactions (DDIs). This comprehensive review explores diverse models and techniques used to assess drug-induced P-gp modulation. We cover several approaches, including *in silico*, *in vitro*, *ex vivo*, and *in vivo* methods, with their respective strengths and limitations. Additionally, we explore the therapeutic implications of DDIs involving P-gp, with a special focus on the renal and intestinal elimination of P-gp substrates. This involves enhancing the removal of toxic substances from proximal tubular epithelial cells into the urine or increasing the transport of compounds from enterocytes into the intestinal lumen, thereby facilitating their excretion in the feces. A better understanding of these interactions, and of the distinct techniques applied for their study, will be of utmost importance for optimizing drug therapy, consequently minimizing drug-induced adverse and toxic effects.

## 1. Introduction

In therapeutics, the simultaneous administration of multiple drugs is very frequent, leading to several drug–drug interactions (DDIs), which may result in a higher or lower bioavailability of a given drug, according to the occurrence of inhibition or induction/activation of drug transporters/metabolic enzymes, respectively [1,2]. By definition, DDIs cause changes in the therapeutic/toxic effects of one compound (substrate) due to the co-administration of another compound (modulator). Moreover, DDIs are triggered by two different types of interactions: at the pharmacodynamic or pharmacokinetic levels. An example of pharmacodynamic interactions is the excessive bleeding observed after the concomitant use of warfarin (a vitamin K antagonist) and low-dose aspirin. By decreasing the production of coagulation factors, warfarin affects bleeding, an effect that is further increased by aspirin through the inhibition of thrombocyte aggregation. On the other hand, pharmacokinetic interactions can also occur due to alterations in the absorption, distribution, metabolism, and elimination (ADME) of a given drug, which may lead to higher or lower drug levels and, consequently, to potential toxicity or loss of therapeutic efficacy, respectively. These alterations may be due to interactions of xenobiotics with drug-metabolizing enzymes, such as those belonging to the cytochrome P450 system (CYP450), involved in phase I reactions, or uridine diphosphate glucuronosyltransferases, involved in phase II reactions [3]. In addition, xenobiotics can also interfere with the transporters present at the cell membranes of different tissues/organs [3,4].

Transporters are large proteins incorporated into the cell membranes, which are expressed in several cells of the major organs with functions of absorption and elimination, such as the liver, intestines, kidneys, brain, testes, and placenta—sites of high importance regarding the evaluation of the pharmacokinetics of xenobiotics [5]. Transporters control the absorption and elimination of xenobiotics and also regulate the movement of small endogenous molecules, such as key metabolites, signaling molecules, vitamins, antioxidants (e.g., uric acid), and some hormones [6]. Recently, the terms phases 0 and III, associated with the uptake and efflux transport of drugs, respectively, were added to the phases I and II that were already associated with the metabolism (functionalization or conjugation, respectively), thus characterizing the complete movement of a molecule in a given cell [7,8]. 

**Figure 1:**
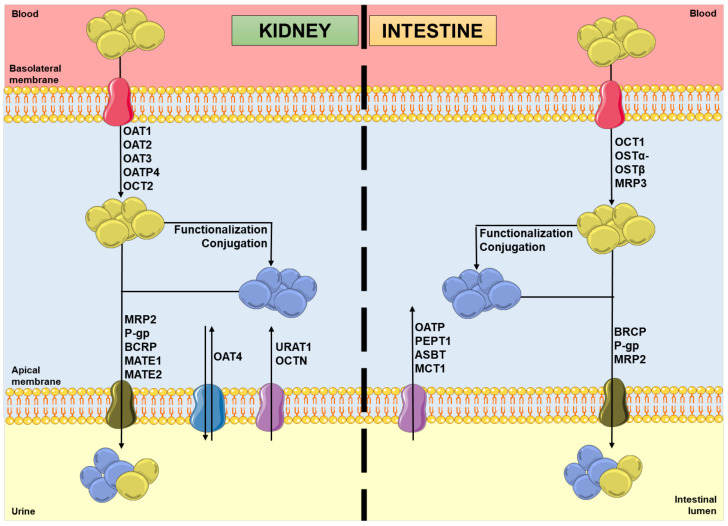
Schematic representation of molecules’ movement and metabolism in proximal tubular epithelial cells and enterocytes, including the transporters’ location and the direction of the substrates’ movement, involved in phases 0 and III of pharmacokinetics. Legend: ASBT: ileal apical sodium/bile acid co-transporter; BCRP: breast cancer resistance protein; blue spheres: xenobiotics; MATE: multidrug and toxin extrusion protein; MCT: monocarboxylic acid transporter; MRP: multidrug resistance protein; OAT: organic anion transporter; OATP: organic anion-transporting polypeptide; OCT: organic cation transporter; OCTN: organic cation/ergothioneine transporter; OSTα-OSTβ: heteromeric organic solute transporter; PEPT: peptide transporter; P-gp: P-glycoprotein; URAT: urate transporter; yellow spheres: xenobiotic metabolites.

In the kidneys, uptake transporters allow the passage of the compounds from blood or ultrafiltrate into the proximal tubular epithelial cells (PTECs), while efflux transporters excrete them back into the blood or into the urine [9]. Furthermore, in the intestines, uptake transporters move the compounds from blood or the intestinal lumen into the enterocytes, while efflux transporters transport them mainly into the intestinal lumen [10,11]. In both organs, there are two superfamilies that represent the most expressed transporters: the solute carrier (SLC) transporters superfamily, which mostly ensures the cellular uptake (phase 0) of compounds from the blood, at the basolateral membrane; and the adenosine triphosphate (ATP)-binding cassette (ABC) transporters superfamily, which enables the efflux (phase III) of their substrates to the tubular/intestinal lumen, at the apical membrane [12,13]. In this way, it is important to note that the tubular secretion of a xenobiotic may involve at least one transporter of each type [13]. Figure 1 shows the major transporters involved in the uptake and/or efflux of substrates at the kidney and intestinal levels. As mentioned above, the xenobiotics’ secretion is based on a coordinated process of transporter-mediated movement across the basolateral and apical cellular membranes. Therefore, the modulation of such transporters by other compounds can potentially result in alterations in the substrate plasma concentrations [14,15].

Despite the undesirable and sometimes life-threatening consequences that DDIs can lead to, on the other hand, it is possible to take advantage of them as a novel therapeutic strategy and, thus, study these transporters as targets to treat diseases [16]. In this sense, the scientific community has made great progress in the predictability and modeling of DDIs [15]. The goal is to evaluate the DDI potential in order to decrease the toxicity risks and/or enhance the therapeutic effectiveness, employing combined approaches using *in silico*, *in vitro*, and/or *in vivo* models [17]. Focusing on the computational models, modeling of transporters may provide important insights into the proteins’ structures, membrane topology, and different conformational states, as well as increasing the knowledge of the substrate binding, translocation, and kinetics processes, and raising awareness of the mechanisms underlying modulators’ modes of action [16].

This review is focused on MDR1/ABCB1, also known as P-gp, given its outstanding role in the detoxification of a broad range of substrates, consequently representing a promising target for mitigating drug-induced toxicity. The processes involved in the modulation of this transporter and the interactions with its substrates in the presence of several compounds (in general, and specifically at the kidney and intestinal levels) will be highlighted and explained in more detail in the following sections. Furthermore, detailed descriptions of the experimental approaches currently used in this field of research for the assessment of P-gp modulation will also be presented. 

## 2. Overview of P-Glycoprotein

### 2.1. Description

P-glycoprotein (P-gp), which comes from “permeability-glycoprotein”, also called multidrug resistance protein 1 (MDR1), is expressed by the *MDR1/ABCB1* gene in humans and belongs to the ABC transporters superfamily, as already mentioned [18,19]. In 1976, Juliano and Ling discovered this protein in hamster ovary cells [20]. Since then, P-gp has been a well-studied protein due to its particularly important role in protecting various sensitive tissues against different xenobiotics, by actively pumping these molecules to outside of the cells and, consequently, decreasing their intracellular concentration and toxicity. Thus, it plays an important role in the process of protection and detoxification, as well as being markedly involved in the phenomenon of multidrug resistance (MDR) in tumor chemotherapy [18,21]. In humans, there are two P-gp isoforms: MDR1 and MDR3. However, only the coding product of the *MDR1/ABCB1* gene actively and significantly participates in the disposition of xenobiotics in various organs. The *MDR3/ABCB4* gene encodes for a phospholipid lipase protein that is mainly expressed on the canalicular domain of hepatocytes, being responsible for the secretion of phospholipids from hepatocytes into bile [22]. In this way, the dysfunction and/or deficiency of this transporter can result in altered phosphatidylcholine–bile salt ratios and, consequently, intrahepatic cholestasis of pregnancy, low-phospholipid-associated cholelithiasis, drug-induced liver injury, or even progressive familial intrahepatic cholestasis type 3 [23]. In this review, we will always be referring only to the P-gp encoded by the *MDR1/ABCB1* gene.

### 2.2. P-gp’s Structure

**Figure 2:**
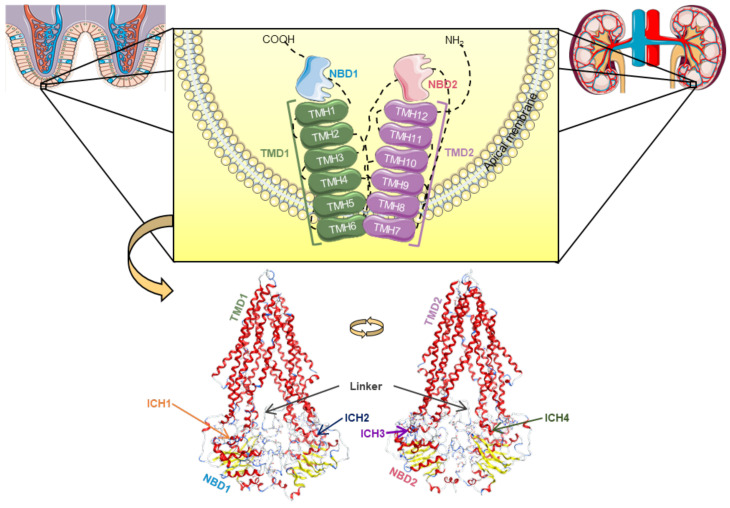
Structural representation of the human P-glycoprotein (P-gp), a full ABC transporter, with the transmembrane domains (TMD1 in green, TMD2 in violet) containing six transmembrane α-helices (TMHs), which are linked to the respective nucleotide-binding domain (NBD1 in blue, NBD2 in pink) by coil bridges between TMH6-NB1 and TMH12-NBD2, and non-covalently by intracellular coupling helices (ICHs): ICH1 (orange)/ICH4 (dark green) and ICH2 (dark blue)/ICH3 (purple) with NBD1 and NBD2, respectively, and both connected by the linker (grey) [29]. The human P-gp model was developed in [29] and adapted to this image using Molecular Operating Environment (MOE) software (version 2019.01).

This transporter is synthesized in the endoplasmic reticulum as a glycosylated intermediate with a molecular weight of 150–170 kDa, with the carbohydrate moiety being further modified in the Golgi apparatus prior to its export to the cell surface [21,24,25]. Like other ABC transporters, P-gp is considered to be a full transporter with two transmembrane domains (TMDs), consisting of six transmembrane helices (TMHs) each, where the substrates connect, and two nucleotide-binding domains (NBDs), where ATP links in order to proceed to its own hydrolysis (Figure 2) [21,26,27,28]. 

Currently, a more detailed constitution of P-gp is accepted among the scientific community, where there are considered to be two homologous functional units (N- and C-terminal halves) with a pseudo-twofold symmetry, each composed of one TMD (comprising six TMHs) and one NBD (containing a catalytic site for ATP binding and hydrolysis). These N- and C-terminal halves are connected by a small peptide sequence (the “linker”), while the TMHs are directly linked to the respective NBD by the intracellular loops, through the functional TMHs 6 (NBD1) and 12 (NBD2), and non-covalently by short intracellular coupling helices (ICHs). These ICHs are located between the structural TMHs 2/3 (ICH1-NBD1), 4/5 (ICH2-NBD2), 8/9 (ICH3-NBD2), and 10/11 (ICH4-NBD1) and have important functions in the maturation and folding of the P-gp transporter, as well as being involved in the signal transmission pathways between the TMDs and NBDs. Furthermore, instead of the two distinct DBSs reported by Shapiro and Ling, (1997), there is now considered to be a large cavity formed by the TMHs of both the N- and C-terminal P-gp halves, called the drug-binding pocket (DBP), which can recognize and accommodate several structurally distinct substrates (Figure 2) [29].

### 2.3. P-gp Efflux Mechanisms

There are two efflux mechanisms proposed for the P-gp-mediated transport: the flippase model, and the hydrophobic vacuum cleaner model [30,31], indicating that the extrusion of the molecules can occur directly from the cytoplasmic compartment or that the protein could act as a membrane pore [31,32]. Currently, the most accepted theory is the “hydrophobic vacuum cleaner” model, which correlates the diffusion of the transported molecules within the inner leaflet of the cellular membrane with interactions at the drug-binding site (DBS) [31,33]. In the late 1990s, Shapiro and Ling proposed the existence of two distinct DBSs, positively cooperating with one another, which were named the H-site and R-site due to the distinct drug specificities registered for Hoechst 33342 and rhodamine-123 (RHO123), respectively [34]. Moreover, it was also shown that molecules could be removed from the inner leaflet to the outer leaflet of the lipid bilayer through both sites, supporting the vacuum cleaner model and postulating the existence of two distinct translocation pathways [31,34,35]. However, several doubts remain today about the specific location of the DBS [31].

P-gp undergoes dynamic conformational changes, through which DBSs alternately access the two sides of the membrane, connecting a charge on one side and releasing it on the other [36,37]. The membrane transporter’s work depends on the energy resulting from the ATP hydrolysis. It has been suggested that the binding of ATP to the NBD and the dimerization of the NBD are driving forces for this function. The hydrolysis energy causes complete conformational changes in the TMD and catalyzes the efflux of the substrate through the TMD and the lipid bilayer. Thereafter, a redefinition of the membrane transporter to its original conformation occurs, allowing new catalytic cycles to start [33,36]. However, the mechanism of communication between the DBS and NBD, along with the extent of the conformational changes leading to the efflux mechanism, remains not fully understood. Although the crystallographic structure of P-gp can be used as a framework to allow a better understanding of its mechanism of action, it has been proven that this study is challenging, significantly decreasing the number of available structures in the Protein Data Bank (PDB) [33].

This transporter exports a broad range of structurally unrelated compounds, being a non-specific transporter [26,29]. P-gp shares some substrates with other ABC transporters, including multidrug resistance protein 1 (MRP1) and multidrug resistance protein 2 (MRP2), more specifically cytostatic agents [26]. P-gp substrates vary greatly in size, structure, and function, ranging from small molecules, such as organic cations, carbohydrates, amino acids, and some antibiotics, to macromolecules, such as polysaccharides and proteins, which is consistent with the knowledge that P-gp is a protective protein against several compounds [24]. Furthermore, several drugs used in therapy are considered to be P-gp substrates, including anticancer agents, cardiac glycosides (e.g., digoxin), β-adrenoceptor antagonists, Ca^2+^ channel blockers, HIV protease inhibitors, steroids, immunosuppressants, antiemetic drugs, antibiotics, antimicrobials, antiretrovirals, and histamine H1 receptor antagonists [7,13,24,38,39]. 

### 2.4. P-gp Expression

P-gp has low expression in most human tissues, but it is found at much higher expression levels in epithelial cells of the intestine (apical membrane of the enterocytes in the lower gastrointestinal tract—jejunum, duodenum, ileum, and colon), limiting the absorption of drug substrates from the gastrointestinal tract; bile ducts of the liver and proximal kidney tubules, resulting in improved excretion of drug substrates into the bile and urine, respectively; the blood–brain barrier (BBB), limiting the entry of a variety of xenobiotics and/or their metabolites into the central nervous system (CNS); hematopoietic cells (where its function remains unknown); and other sites, such as the pancreatic ducts, adrenal glands, placenta, endometrium, and testicles. In general, the location of this protein reinforces the accomplishment of its protective/barrier function. Its physiological role in secreting endogenous and exogenous compounds (through phase III-mediated transport) influences the (pharmaco)toxicokinetics of several compounds [21,22,25,40]. This is what happens in the so-called “normal” cells under physiological conditions. However, in tumor cells, there is frequently an overexpression of P-gp, which causes anticancer agents to be exported from the tumor cells. This phenomenon keeps the concentrations of these drugs below the therapeutic threshold, which can lead to a subtherapeutic effect on these cells, thus creating tumor resistance to these types of drug agents [36]. 

Additionally, polymorphisms affecting the *MDR1/ABCB1* gene should also be considered. Indeed, *MDR1/ABCB1* polymorphisms are associated with lower renal digoxin clearance, as well as modified efficacy and nephrotoxicity of chemotherapeutics, antivirals, and immunosuppressants, like cyclosporine (due to impaired apical efflux) [13]. In addition to these effects, *MDR1/ABCB1* polymorphisms can also contribute to the alteration of the drugs’ disposition and, consequently, to interindividual variability in response to several drugs, like morphine and other opioids (e.g., methadone) [41]. Some authors have studied the polymorphisms of the P-gp-coding gene. Hoffmeyer et al. observed a significant correlation between a P-gp polymorphism (C3435T in exon 26) and the levels of P-gp’s expression and function. Individuals who were homozygous for this polymorphism (24% of the sample population (*n* = 5188)) had significantly lower duodenal P-gp expression and the highest digoxin plasma levels, suggesting that this polymorphism may affect the absorption and tissue concentrations of numerous other P-gp substrates [42]. In addition, Saiz-Rodríguez et al. performed clinical trials with 473 healthy volunteers to analyze the C3435T polymorphism, concluding that it can affect the elimination of some drugs in different ways. They observed an enhanced elimination of risperidone, trazodone, and dehydroaripiprazole, while in the cases of olanzapine and citalopram a reduction in their elimination occurred. On the other hand, there were no changes detected in the elimination of quetiapine, aripiprazole, sertraline, or agomelatine [43]. Kim et al. carried out a polymorphism analysis of 37 healthy European-American and 23 healthy African-American subjects, identifying 10 single-nucleotide polymorphisms (SNPs). Three SNPs (C1236T in exon 12; C3435T in exon 26; G2677T, Ala893Ser in exon 21) were found to be linked (MDR1*2 allele, which is suggested to cause enhanced P-gp activity) and occurred in 62% of European Americans and 13% of African Americans. *In vitro* experiments indicated an enhanced efflux of digoxin (50 µmol/L) from cells expressing the MDR1-Ser893 variant (HEK293T cells, human embryonic kidney 293 (HEK293) containing simian vacuolating virus 40 (SV40) large T antigen). In humans, MDR1*1 and MDR1*2 variants were associated with differences in the plasma levels of fexofenadine (a known substrate of P-gp); these results were consistent with the *in vitro* data, suggesting that, *in vivo*, P-gp activity in subjects with the MDR1*2 allele is also enhanced [44].

Therefore, a deeper understanding of P-gp’s structure and efflux transport mechanism is fundamental for investigating the phenomena of modulating the activity of this transporter and the consequent modification of the (pharmaco)toxicokinetics of several xenobiotics [29].

## 3. Study Models of Drug–Drug Interactions Involving P-Glycoprotein Modulation

P-gp modulation could be a problematic strategy of therapy, especially when its substrates are drugs with a narrow therapeutic index, leading very promptly to toxicity or a lack of therapeutic effect [45]. In this way, several agencies, like the European Medicines Agency (EMA) and the U.S. Department of Health and Human Services Food and Drug Administration (FDA), recommend the assessment of interactions between P-gp and all new investigational drugs [46,47]. There is a great diversity of studies using different types of assays and models, including cell cultures, tissues, and even animal and human experimentation, as well as computational simulations. All of these studies have advantages and limitations. For instance, in the field of biology, *in vitro* models are commonly employed. Nevertheless, this poses challenges in pinpointing the precise targets of chemical attacks and defining the endpoints, as well as extrapolating the results to the human body. This complexity adds to the difficulty of interpreting the data accurately. *In vivo* studies provide a better perspective on the (pharmaco)toxicokinetic characteristics and the consequent extrapolation to the human organism. However, from an ethical point of view, they do not provide easy conditions to carry out larger screenings of compounds and/or experimental conditions [48]. Nevertheless, it should be noted that due to the lack of standardization, and to the existence of contradictory data, the interpretation of the results reported in the literature can be very difficult [45]. For the evaluation of transporter activity and the respective interactions with new compounds, all types of studies should be carried out in a complementary manner, giving more reliable results. Table 1 highlights the main advantages and limitations of the different research models used in the assessment of P-gp modulation. Accordingly, in the following sections, the diverse *in silico*, *in vitro*, *ex vivo*, and *in vivo* models/assays and clinical trials used in this field of investigation will be further discussed in more detail. 

### 3.1. In Silico Models

Computational models are valuable tools for the simulation of biological systems, and a reliable alternative to excessive animal use. In this way, molecular docking analysis can be used as an initial approach to perform larger and faster screenings to identify P-gp modulators/substrates, instead of the experimental techniques, which are more expensive, laborious, and time-consuming [33,49]. Nevertheless, these techniques present some limitations that complicate the selection of the technique that should be used, such as the following: (a) inhibition assays are not generally predictive of substrate transport, and vice versa, which makes the selection of the method an important phase in each study; (b) the substrate can be a weak inhibitor, depending on the strength of its molecular interactions with the transporter-binding site, and inhibitors can also be transported; (c) allosteric binding sites and/or transport modes are another source of complexity in this type of analysis, resulting in the existence of multiple models with multiple binding sites and allosteric mechanisms of interaction; (d) the substrate can be transported by one or more transporters; (e) the transported molecule is typically the result of multiple parallel processes, including varying diffusion rates across the lipid bilayers [16].

Several studies performed *in silico* have clarified the stability of P-gp’s molecular structure, along with its mechanisms of action, substrate binding, or conformational changes, using ligand-based molecular modeling (including the use of pharmacophores), quantitative structure–activity relationship (QSAR) analyses, and molecular dynamics simulations [33,49,50].

Ligand-based molecular modeling refers to the interactions between compounds and the transporter, correlating the molecular properties and structure of a set of ligands with a given transporter and its respective activity. Models are typically developed based on previous data and then used to predict the activity of other ligands (considering their molecular properties and structure), or even the structure of the transporter. However, several factors of the surrounding environment must be determined, typically using specialized pre-filtering software, including the ionization state, stereochemistry, and absence/presence of counterions [16]. Ligand-based molecular modeling approaches include Hansch analysis, (non)linear classification algorithms, (un)supervised artificial neural networks, and pharmacophore modeling [51]. Pharmacophore modeling is a 3D molecular modeling approach that uses a ligand-based pharmacophore to describe distinct pharmacophoric molecular properties, such as hydrogen-bond acceptors, hydrogen-bond donors, hydrophobic features, and negatively and positively ionizable atoms, which are essential to be identified by the transporter [16]. That is, the pharmacophore model defines the minimum structural characteristics that a molecule must have to bind to P-gp [52]. Palmeira et al. developed a pharmacophore model from 26 flavonoids known to be P-gp inhibitors, which was then used to screen the DrugBank platform. With this *in silico* model, the researchers were able to select 21 compounds for laboratory testing, 12 of which proved to be P-gp inhibitors [53].

With the first X-ray structures obtained, the computational studies evolved to structure-based studies [51]. Structure–activity relationship (SAR) analysis determines the level of influence that the structural alterations of individual molecules have on their modulation effect [52]. On the other hand, QSAR can quantify the interactions between the molecular descriptors and P-gp activity. That is, QSAR models use physicochemical descriptors and connectivity/structure fingerprints (numerically, in two dimensions (2D)) to predict the effect of a ligand on the transporter activity via mathematical models [16,52]. In this case, the physicochemical descriptors include non-covalent interactions (e.g., lipophilicity, hydrogen bonding, charge, aromaticity), H-bond acceptor strength, and other spatial properties (e.g., molecular size, flexibility, polar surface area, atomic connectivity) [16,51,54]. Also, the models can vary from simple linear equations to complex multivariate and nonlinear statistical models. This type of model allows molecules to be tested for their ability to modulate the transport or be transported by a specific transporter of interest. Then, molecular descriptors from the library of tested molecules are used and, as a result, the model is generated using statistical modeling algorithms [16]. For example, this technique was used by Ghaemian and Shayanfar with the aim of predicting the P-gp-inhibitory activity of epigallocatechin and gallocatechin derivatives. They were able to calculate the pixels of images and their principal components and then develop QSAR models using different approaches, such as principal component regression, artificial neural networks, and support-vector machines. All of these approaches were able to predict the inhibitory effects caused by epigallocatechin and gallocatechin derivatives. However, the artificial neural network produced the best results [55]. There are still ligand modeling techniques in 3D, such as 3D-QSAR, which considers descriptor-based QSAR models with spatial 3D molecular structures of ligands and their relationships with transporter activity. In this technique, the biological activity of the transporter is correlated with the non-covalent interactions occurring in the environment surrounding the molecule, like steric fields (based on the shape of the molecule) and electrostatic fields (e.g., van der Waals and electrostatic forces) [16,52].

It should also be noted that, throughout the years, others approaches have been developed, like Hansch and Free-Wilson analyses, hologram QSAR, comparative molecular field analysis, comparative molecular similarity index analysis, nonlinear methods, and similarity-based approaches [54]. The use of a set of 22 new chalcone derivatives, synthetized and classified as P-gp inhibitors *in vitro* (CEM.VCR1000 cell line), allowed for the development of a 3D-QSAR model that could be further used in the screening of potential P-gp modulators. The 3D structures were built, their energy was minimized using Molecular Operating Environment (MOE) software, and the alignment-independent 3D descriptors were computed using Pentacle (version 1.06) software to retain the information encircled in the molecular interaction fields and other variables independent of the spatial position. Later, they calculated the interaction energy, which includes Lennard–Jones energy, hydrogen bonds, and electrostatic interactions. These 3D-QSAR studies indicated that P-gp inhibition is influenced by H-bond acceptors, methoxy groups, hydrophobic groups, the number of rotatable bonds, and pharmacophoric features [56].

Molecular dynamics simulations are another *in silico* approach that can be applied in DDI research, evaluating the structural dynamics of P-gp at high spatial and temporal resolution. In other words, molecular dynamics techniques enable researchers to observe the conformational changes of P-gp during its transport process, at the atomic level. P-gp is a very flexible transporter with several conformations, probably related to its polyspecificity (i.e., the ability to bind to several chemically diverse compounds) [57,58]. Using hydrogen–deuterium exchange mass spectrometry (HDX-MS), Kopcho et al. successfully analyzed P-gp’s dynamics in three conformational states: predominantly inward-facing apo P-gp, pre-hydrolytic P-gp bound to Mg^2+^-ATP, and outward-facing P-gp bound to Mg^2+^-ADP-VO_4_-3. The authors concluded that their findings suggested an asymmetry between the NBDs and, additionally, that a pre-hydrolytic P-gp state occurs in an occluded conformation [59]. Lagares’ group demonstrated that NBDs’ conformational distribution/dynamics will differ in the presence of (non)active compounds within the DBP, which could affect the patterns of movement and structural variations, leading to an activation of the transport process [58]. Dehghani-Ghahnaviyeh and coworkers also used molecular dynamics simulations to evaluate the NBD sites and other conformational alterations in multiple nucleotide-binding states, suggesting that both global and local conformational alterations that occur in NBDs are due to ATP hydrolysis and nucleotide dissociation [57]. 

**Figure 3:**
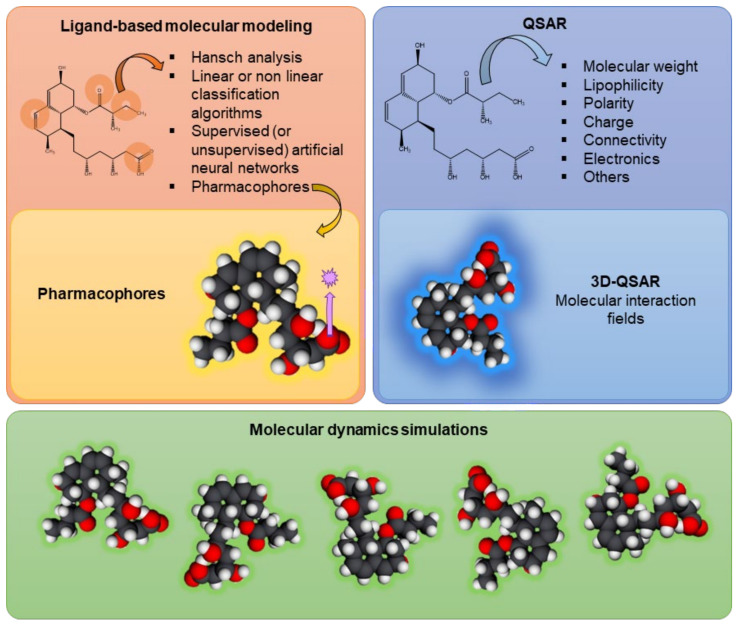
Schematic representation of *in silico* techniques currently used in the evaluation of drug–drug interactions at the P-glycoprotein level, such as ligand-based models, including the use of pharmacophores; quantitative structure–activity relationship (QSAR) models, including 3D-QSAR; and molecular dynamics simulations.

Figure 3 summarizes the *in silico* models currently used in the assessment of P-gp modulation, including ligand-based models, QSAR models, and molecular dynamics evaluations. 

All of the models described above are used in the evaluation of P-gp modulation and can be mostly divided into four groups: (a) development/optimization of P-gp structures, (b) generation of datasets of P-gp substrates, (c) evaluation of P-gp inhibition/generation of datasets of P-gp substrates, and (d) assessment of P-gp induction and/or activation/generation of datasets of P-gp inducers and/or activators.

In 2006, Vandeveur and coworkers presented a 3D atomic model of P-gp, obtained from a combination of comparative modeling and rigid body dynamics simulations characterizing the efflux pump in the absence of ATP. In agreement with electron microscopy and with the experimentally acquired data, the model indicated the possibility of multiple DBSs, previously documented interactions (including H bonds and cation interactions), and a lateral gap in the TMD (connecting the lipidic bilayer to the P-gp’s central cavity) [60]. 

Ferreira and coworkers’ group had also been working in this field, starting with the creation of a human crystallographic structure of P-gp. They evaluated the stability of the crystallographic structure, observing TMD disorganization and NBD separation in the absence of a lipid bilayer, which led to an irreversible distortion of the P-gp structure. Thus, the importance of the inclusion of the protein in the lipid bilayer and the presence of the linker sequence was determined [33]. Subsequently, Bonito et al. generated a human P-gp homology model, using the murine P-gp crystallographic structure of 2015 as template (PDB ID: 4Q9H). They selected this murine crystallographic structure template for three reasons: (a) this was the most recently published crystallographic structure of murine P-gp; (b) upgrades were made to the resolution of several TMHs, ICH1, and some extracellular loops, showing that it is a reliable human P-gp model; (c) the absence of ligands or antibody complexes that could have any influence on the TMD’s native arrangement. Therefore, this template was inserted in a suitable lipid bilayer, enhanced through molecular dynamics simulations, and then thoroughly validated [29,61]. The validation of protein models can be performed by using several software platforms, including MOE, the Swiss-Model online server, and the analysis of Ramachandran plots [31,33,62].

Since the determination of P-gp’s structures, one of the priorities of researchers in the evaluation of DDIs has been to generate datasets of compounds involving P-gp modeling for further investigations, usually performed using ligand-based models or through the validation of structure-based virtual screening [16]. There are several databases available for the collection/entry of data when researchers are building these datasets, such as ChEMBL, PubChem, and MEDLINE [51].

Several types of studies have long been used to simulate the ligand−protein pocket interactions at the P-gp level. In 2003, Didziapetris et al. introduced the “rule of fours”, where compounds with ≥8 N and O atoms, molecular weight (MW) > 400, and acid pKa > 4 were expected to be P-gp substrates, while compounds with ≤4 N and O atoms, molecular weight <400, and base pKa < 8 were probably non-substrates [63]. In this way, it was established that compounds are defined as modulators or substrates according to the number of interactions created within the DBS. That is, modulators frequently establish a higher number of simultaneous interactions [33]. 

Several other techniques can be used to perform this evaluation. Gombar et al. developed a QSAR model through the evaluation of a training set with 95 compounds used *in vitro*. The QSAR model was a two-group linear discriminant model that used 27 statistically significant information-rich structure quantifiers to characterize compounds as substrates or non-substrates (with an accuracy of 86.2%) [64]. Using binary classification algorithms (substrate or non-substrate?), Li et al. compiled information about 423 substrates and 399 non-substrates, showing that molecular weight and solubility (of the eight physicochemical proprieties evaluated) are the major properties differentiating substrates from non-substrates (i.e., P-gp substrates have higher molecular weights and tend to be less soluble). Additionally, the researchers carried out a comparative study between the 423 substrates and 735 inhibitors, concluding that the inhibitors were significantly more hydrophobic, while the substrates tended to have more H-bond donors [65]. 

Using pharmacophores, another group was capable of identifying hydrophobic and hydrogen-bond acceptor groups (the main groups established within the DBS), as well as the motion patterns of P-gp, analyzing the motions in a 100 ns simulation (POPC-II), allowing for the detection of the major motion patterns in the absence of substrates [33]. Coordinating their research with *in vitro* assays, Desai et al. developed a QSAR model with positive and negative prediction values exceeding 80%, based on information obtained from structurally diverse data for more than 2000 compounds, showing once again that *in silico* and/or *in vitro* techniques are valuable tools in the beginning of drug discovery research [66]. Along with *in vitro* assays, other authors performed molecular docking, followed by molecular dynamics simulations to analyze the identified drug–transporter interactions more mechanistically, showing that darunavir and dapivirine were P-gp substrates, while tenofovir presented a lower free binding energy [67]. More recently, researchers developed an *in silico* model to evaluate the brain-to-plasma concentration ratio (K_p,brain_) and unbound brain-to-plasma concentration ratio (K_p,uu,brain_) of P-gp substrates, validated by newly acquired experimental brain penetration data of 28 P-gp substrates. Thus, the proposed method was suggested for the drug discovery field related to the CNS, not only to predict the pharmacological effects, but also to improve new drugs [68].

Several *in silico* models are freely available online. Guéniche and coworkers performed a comparative study of six freely available web tools (ADMETlab, AdmetSAR2.0, PgpRules, pkCSM, SwissADME, and vNN-ADMET) using a set of 231 FDA-approved drugs (2010–2020) characterized *in vitro* as P-gp substrates. Whether the web tools were used alone or in combination, the compounds were found to weakly meet the criteria commonly accepted for prediction, which warn researchers to use these online *in silico* models with caution [69].

As already described, P-gp has been seen as a potential therapeutic strategy, making the search for P-gp modulators a goal in several investigations. First, the evaluation of P-gp inhibitions as a strategy to overcome the MDR phenomenon was considered worldwide, being reported in several studies. Using a murine P-gp structure, Wang and coworkers built a QSAR model based on a Bayesian regularized neural network compiling a dataset of 57 flavonoids described as inhibitors [70]. Ferreira and coworkers reported a modulator site (so-called M-site) in addition to the two DBSs identified by Shapiro and Ling: the H-site and the R-site [34], characterized by cross-interactions between both P-gp halves, with an important role in impairing conformational changes leading to substrate efflux. The DBS characterization demonstrated that the M-site and the H/R drug-binding sites can be differentiated by their amino acid composition. After the characterization of the interactions taking place in the DBP, it was possible to classify the compounds as modulators or substrates based on a database analysis [31]. In the same year, Jara et al., by performing molecular docking and dynamics simulations in a crystallographic structure of murine P-gp, reported that hydrophobicity and molecular flexibility were the main proprieties related to the inhibition process [71]. Using a human structure of P-gp obtained from molecular dynamics studies, Brewer and coworkers presented a ligand docking technique useful to discover reversible inhibitors of ATP hydrolysis, connecting with the NBDs. The authors’ results suggest that this mechanism should be evaluated specifically in the NBDs [72].

Along with *in vitro* evaluations, Morsy et al. evaluated the P-gp inhibition process using a human crystal structure (PDB: 2CBZ) and MOE and Triangle Matcher software. Among 12 naturally occurring compounds, piperine had the higher binding affinity, which was further proven *in vitro* by the researchers [73]. In another study, *ab initio* simulation and molecular docking studies of P-gp’s interactions with saxitoxins and lipoic acid showed that saxitoxins can be P-gp substrates, while lipoic acid is probably an inhibitor (fewer amino acids remained in the DBS, like verapamil). Further *in vitro* evaluations complemented these findings, demonstrating a reduction in saxitoxin-induced cytotoxicity in the presence of lipoic acid [74]. Molecular dynamics studies helped Mollazadeh and coworkers to investigate the inhibitory effects of 1,4-dihydropiridine on P-gp activity, demonstrating the high binding affinity between a derivative of this compound and the DBS on a murine crystallographic structure (PDB: 3G60), in agreement with the *in vitro* assays performed [75]. Later, they also evaluated how both symmetric and asymmetric (R,S)-1,4-dihydropiridine interacts with P-gp, using biological assays, molecular docking, and molecular dynamics simulations, showing that stereochemistry cannot be neglected during the development of these new P-gp modulators [76].

The direct evaluation of the interaction between P-gp and the natural sesquiterpenes β-caryophyllene and β-caryophyllene oxide was performed by molecular docking and dynamic simulation studies, along with the *in vitro* evaluation of these compounds’ effects on doxorubicin-induced cytotoxicity. The authors found that caryophyllene sesquiterpene binds next to the ATP-binding domain, while β-caryophyllene has a higher binding affinity and, consequently, a higher inhibition potential. *In vitro* studies performed with human hepatoma cells (HepG2 cells) demonstrated an increase in the cytotoxicity caused by doxorubicin (0–100 µM for 24, 48, or 72 h) in the presence of β-caryophyllene (50–100 µM for 24, 48, or 72 h) [77]. Júnior et al. studied the P-gp (and CYP450)-inhibitory effects caused by extracts from *I. setifera* (dry season) and *I. asarifolia* (dry and wet seasons). Using a machine-learning-based algorithm, they found the possible constituents responsible for the toxicity of the plant extracts: caryophyllene oxide and cedroxyde, probably due to presence of epoxide in their structures; and phytol, due to the inhibition of the P-gp and CYP450 enzymes, which leads to increased absorption of the essential oils of the plant [78].

Durães et al. designed and synthesized a library of thioxanthones (TXs) as potential efflux pump inhibitors. *In vitro*, in Caco-2 cells, a lack of cytotoxicity of the developed compounds was demonstrated for concentrations up to 20 µM and 24 h after exposure. Furthermore, their potential for P-gp modulation was also confirmed. Indeed, one of the tested compounds (compound 14; 20 µM) significantly decreased P-gp expression 24 h after exposure, and two of the tested TXs (compounds 12 and 13; 20 µM) promoted an immediate decrease in P-gp activity after a short period of incubation (90 min). In the docking studies, the aim was to predict the binding affinities of a library of 13 thioxanthone derivatives and 4 tetracyclic thioxanthenes against a homology model of mammalian P-gp, at two different sites: the TMD and NBD. The molecules in the study presented docking scores similar to those of known P-gp inhibitors (as reported in the literature) [79]. 

Pitsillou and coworkers reported a study on the assessment of the ADME and toxicity profiles of 675 compounds from OliveNet™ by molecular dynamics. Of the studied compounds, 313 were expected to be absorbed by the gastrointestinal tract, among which hydroxytyrosol needed the least force to pass through the lipid bilayer. The authors suggested further *in vitro* evaluation for P-gp’s inhibition of the following potential candidates: oleuropein aglycone decarboxymethyl dialdehyde acetal form, decarboxymethyl elenolic acid dialdehyde, acetal of decarboxymethyl elenolic acid dialdehyde, methyl malate-β-hydroxytyrosol ester, hydroxytyrosil elenolate, d-(+)-erythro-1-(4-hydroxy-3-methoxy)-214-phenyl-1,2,3-propantriol, (+)-1-acetoxypinoresinol-4-*O*-methyl ether, and 3-[1-(hydroxymethyl)-(*E*)-1-propenyl] glutaric acid, due to their properties (e.g., good absorption and membrane permeability, non-hepatotoxicity, non-substrates of P-gp, and non-inhibition of CYP450 enzymes or hERG) [80]. 

On the other hand, several researchers have been studying the possibility of induction/activation of P-gp (e.g., TXs, flavonoids) as a new potential therapeutic strategy, developing several *in silico* models in this field. Along with the *in vitro* assays, Silva and her group investigated the interactions between P-gp and colchicine, an alkaloid derived from the *Colchicum autumnale* family of plants. According to the *in vitro* results, the researchers developed two 3D pharmacophore models for newly synthesized thioxanthonic derivatives (23 noncompetitive and 19 competitive P-gp inhibitors). Using the Catalyst program, it was possible to generate the numbers of conformers related to the luminescence values obtained from ATPase assays. The selected models were validated with a set of 11 known P-gp inhibitors and then used as a query for colchicine mapping. Using the “Best Fit” method in Catalyst, they performed a flexible fitting process, analyzing the fit scores. Also, docking studies were performed in the DBP to discover the binding affinity of colchicine. To assess these DDI evaluations, the researchers used AutoDock Vina, with the target conformation as a rigid unit and colchicine flexible and adaptable to the protein, and PyMOL to visualize the different interactions and conformations of the protein. They observed, *in silico*, that colchicine has a competitive mechanism of transport, since it was expected to bind to the DBP of P-gp. In this way, in agreement with the results obtained from *in vitro* studies, *in silico* results suggested that, although colchicine is a P-gp inducer, it also acts as a competitive P-gp inhibitor [81]. Wongrattanakamon et al. developed a multivariate linear QSAR model to predict the modulatory effects of compounds on P-gp, applying 23 bioflavonoids as a training set, including the inhibition and induction processes. After the model’s validation, the authors used an external set containing 11 flavonoids, obtained from the literature, to test the model. It was observed that the *in silico* model was capable of classifying the modulatory activities of seven flavonoid compounds: naringenin, quercetin, morin, EGCG, ECG, biochanin A, and hesperidin [82].

Regarding the *in silico* evaluation of P-gp activation, the published research is very scarce, to best of our knowledge. In accordance with the *in vitro* activation results obtained with thioxanthonic derivatives, Silva and coworkers developed a pharmacophore model for P-gp activation, aiming to further screen for new P-gp activators. The researchers used a set of 19 known P-gp activators as active ligands to create common feature pharmacophore models. Additionally, they set the minimum features to 1, the maximum features to 10, the conformation generation to “BEST”, the “Maximum Omitted Features” to 0, and even generated 255 different conformations per molecule. The generated pharmacophore was successfully validated using a test set of eight known P-gp activators, using Catalyst software (Accelrys 2.1) [83].

Therefore, analyzing all of these studies, we can see that *in silico* techniques are an important way to complement and/or help researchers in starting a new project, or to improve their understanding of the results obtained from other laboratory methodologies for a deeper elucidation of the drug-mediated P-gp modulation. One great example is BINding ANAlyzer (BINANA) 2.0, an emerging software platform that allows researchers to identify, characterize, and visualize receptor–ligand complexes, including the specific P-gp residues to which compounds bind. Therefore, BINANA 2.0 can be a great tool to inform researchers on which P-gp modulators they should start their investigations or, on the other hand, to acquire a better understanding of the experimental results already obtained [84].

### 3.2. In Vitro Models

Focusing on the experimental laboratory models, *in vitro*, *ex vivo*, and *in vivo* methods are crucial for drug discovery research. Nevertheless, when it comes to these types of studies, the proper selection and application of relevant models is of paramount importance, as is the accurate interpretation of the obtained results. In fact, transporters have been extensively studied through *in vitro* methods, which can provide valuable insights into the interactions of compounds with their respective transporters [85]. *In vitro* techniques offer several advantages, including being less expensive and time-consuming, allowing for the assessment of a high number of compounds simultaneously, evaluating both expression and activity, and not being subject to ethical restrictions. These advantages are particularly important in this area of research, because relying on a single method or evaluating only one compound can potentially lead to false results. On the other hand, the use of blanks, as well as positive and negative controls, is also noteworthy. Regardless of the method used, the most common practice is to expose the cells to the toxic P-gp substrate, in the presence and absence of the potential modulator, and then evaluate the significant differences in cytotoxicity/fluorescence in its presence/absence [38]. Additionally, it should also be noted that different cell lines express different amounts of transport proteins, which will lead to different outcomes in these types of studies. Shapiro and Ling found that the P-gp-mediated transport of Hoechst 33342 was directly proportional to the cell concentration and inversely proportional to the aqueous concentration [34] and, as such, paved the way for different studies conducted in this regard [86]. 

**Figure 4:**
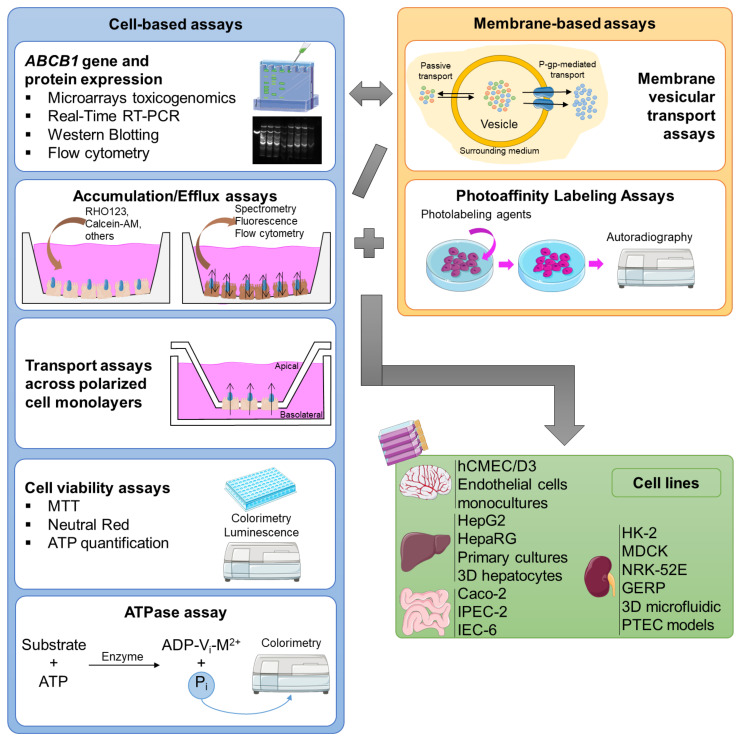
Representative scheme of the major cell-/membrane-based models and the major cell lines used for the *in vitro* evaluation of P-glycoprotein modulation.

The *in vitro* assays can be divided into two major groups: cell-based assays, and membrane-based essays, each applied to the type of cell for which it is most adequate for the specific study (Figure 4).

Cell-based assays can offer clear-cut information about the interactions between compounds and the transporter, and they can be applied in the assessment of K_m_ (substrate concentration at half-maximum velocity (V_max_)) and V_max_ (maximum velocity), as well as K_i_ (the disassociation constant for an inhibitor–P-gp complex) and IC_50_ (half-maximal inhibitory concentration), for substrates and inhibitors, respectively. Therefore, the use of the Michaelis–Menten steady-state formula (Theorem 1) can be very useful for estimating kinetic parameters, such as K_m_ and V_max_, for carrier-mediated transport. Additionally, IC_50_ and K_i_ have also been determined with the aim of estimating P-gp inhibition in order to predict the saturation and inhibition of P-gp and other transporters *in vivo* [86].

$$V_{0}=\frac{V_{max}[S]}{K_{m}+[S]}$$

**Theorem** **1.** *Michaelis–Menten equation. Legend:* V_0_*: velocity of transport;* [S]*: substrate concentration;* V_max_*: maximum velocity;* K_m_*: substrate concentration at half* V_max_*; Michaelis constant.*

In these types of studies, cells are seeded in multiwell plates, allowing for the investigation of several conditions, concentrations, and/or exposure times in a single plate. Nevertheless, they can be more labor- and time-consuming than the membrane-based assays [38]. Currently, the major cell-based assays used in the assessment of P-gp modulation are as follows:

***ABCB1* gene expression:** Northern blotting analysis, dot blot analysis, competitive polymerase chain reaction (PCR), RNAse protection assays, and *in situ* hybridization, techniques capable of assessing changes in genes’ location/expression in tissues, are widely accepted by the scientific community for the evaluation of transporters’ gene expression. In particular, real-time (RT)-PCR is one of most used techniques for the analysis, detection, and quantification of mRNA, with the assistance of fluorescent probes and glyceraldehyde 3-phosphate dehydrogenase (GAPDH) as a reference gene in human tissues (alternative reference genes can be used for other cell systems). This sensitive technique automatically enables the use of multiwell plates and, consequently, enables high-throughput screening [38].

**Microarray-based toxicogenomics**: Using gene arrays, alterations in gene expression induced by classical toxic compounds, functional genomic profiles, and mechanistic markers of toxicity are investigated. Afterwards, the profiles of known toxic agents are used to compare the gene expression signatures of the potential candidates [87].

**Western blotting:** Also called immunoblotting or protein blotting, this permits the detection of proteins (post-electrophoresis), even when in low quantities. This method is frequently used in drug discovery research, with the aim of evaluating the transporters’ protein expression, which can be assessed in the presence and absence of modulators. In this field, it is notable that it is possible to obtain several replicas of the same gel, allowing for multiple analyses [38].

**Flow cytometry:** Flow cytometry offers a complete cellular analysis (e.g., cell size and internal complexity) by measuring their optical and fluorescence characteristics. With the use of fluorescent antibodies, it can be an important tool for understanding the regulation and interaction of cell systems, namely, the evaluation of P-gp expression. Although propidium iodide, phycoerythrin, and fluorescein are the most used dyes, the use of other fluorescent compounds that are also P-gp substrates permits the assessment of the transporter activity [38]. Among others, RHO123, DiOC2_(3)_, calcein-acetoxymethyl ester (calcein-AM), doxorubicin, daunorubicin, or mitoxantrone can be used [88].

**Accumulation/efflux assays**: These methods are based on exposing the cells under study to fluorescent P-gp substrates, which depend on this transporter for export. Therefore, the accumulation of these fluorescent substrates will depend on the activity of the transporter, and it is possible to compare it in the presence and absence of potential modulators. In other words, the intracellular accumulation of a fluorescent P-gp substrate will be inversely proportional to the P-gp activity and can be easily measured by spectrophotometry (or by flow cytometry, as mentioned above). The most used assays reported in the literature are the RHO123 accumulation assay and the calcein-AM assay [38]. Calcein, a fluorescent compound, is accumulated inside the cells after ester hydrolysis of calcein-AM, its non-fluorescent precursor. This assay is based on the principle that only calcein-AM is exported by P-gp and, for that reason, the measurement of the intracellular accumulation of calcein indicates the level of P-gp transport activity [89]. 

The accumulation/efflux assays, with or without flow-cytometry-based approaches, are the most used assays in the evaluation of P-gp’s function and the respective drug-mediated modulation. Silva et al. studied the ability of several compounds to modulate P-gp expression and activity using a flow-cytometry-based approach. The researchers used the UIC2 monoclonal antibody conjugated with fluorescein isothiocyanate to assess P-gp expression, and they used RHO123’s intracellular accumulation to assess P-gp activity. This antibody is a preferable choice due to its capacity to detect an external epitope of P-gp, enabling the detection of the protein already incorporated into the cell membrane [90,91]. The fluorescence of the fluorescent P-gp substrate and of the UIC2 monoclonal antibody was then measured by flow cytometry, with or without exposure to the test compounds. With the aim of assessing the influence of aging on the expression and function of P-gp in human lymphocytes, Vilas-Boas and coworkers assessed the P-gp expression/activity in lymphocytes isolated from whole-blood samples of 65 healthy Caucasian male donors, using the UIC2 antibody conjugated with phycoerythrin (PE) to evaluate P-gp expression and, on the other hand, using the RHO123 accumulation and UIC2 shift assay to assess P-gp activity. The UIC2 shift assay was performed using the monoclonal antibody against the extracellular conformational epitope of P-gp, UIC2, in the presence and absence of vinblastine, and is based on the UIC2 shift phenomenon: under physiological conditions, the reactivity of UIC2 will be increased in the presence of P-gp substrates. The fluorescence of the PE bound to the UIC2 antibody was then measured by flow cytometry. No alterations were observed between the results obtained from the RHO123 accumulation and UIC2 shift assays with respect to P-gp activity. On the other hand, the results indicated a significant age-dependent increase in P-gp expression (particularly in men older than 60 years), although no differences were observed in P-gp activity [92]. 

**ATPase assays:** ATPase assays measure changes in P-gp ATPase activity in the presence of P-gp substrates or modulators. Therefore, there are two different approaches to carry out the analysis. The substrates/modulators can directly interact with the efflux pump, causing a decreased/increased formation of the complex ADP-V_i_-M^2+^ (where V_i_ is the inorganic vanadate and M^2+^ is a divalent cation). The complex remains linked to one of the NBDs of this pump, leading to an intermediate state of P-gp and disabling the transport. Normally, to evaluate the effect of the substrate/modulator on P-gp’s function, the amount of inorganic phosphate (P_i_) is measured by a colorimetric reaction, being directly proportional to the P-gp ATPase activity. On the other hand, the level of unmetabolized ATP established by a luciferase-generated luminescence signal can also be measured, which is inversely proportional to the P-gp ATPase activity [38].

**Cell viability assays:** Cell viability is a determinant for the number of healthy cells and the cell proliferation in a given sample, and it is based on cell functions like enzyme activity, cell membrane permeability, cell adherence, ATP production, coenzyme production, and nucleotide uptake activity [93]. These assays are particularly useful in this field, not only to evaluate the impact of P-gp on the toxicity of a given substrate, but also to assess changes in the toxicity of a given substrate in the presence of an inhibitor/inducer/activator [81,94]. In this research field, three of the most used cytotoxicity assays are the following: (A) 3-(4,5-dimethylthiazol-2-yl)-2,5-diphenyltetrazoliumbromide (MTT) reduction assay: The MTT reduction assay is based on the principle that metabolically viable cells will reduce the yellow-colored MTT via the action of mitochondrial dehydrogenases, producing purple formazan crystals. By simply measuring the optical purple color, the percentage of metabolically viable cells (directly proportional) is obtained [95]. (B) Neutral red (NR) uptake assay: The vital dye NR is added to the cultured cells previously treated with the compounds under study. Then, the amount of NR retained inside the cells is measured, in the lysosomes, providing a quantitative estimation of the viable cells in the culture, as only viable cells will retain the dye [96]. (C) ATP bioluminescence assay: The ATP quantification assay is based on the emission of light from the reaction between ATP and luciferin, which is catalyzed by the luciferase enzyme. That is, after removing the cell culture medium and lysing the cells, a solution containing d-luciferin is added, which reacts with ATP, generating a luminescent light. Thus, using the luminescence technique, the intracellular ATP levels are measured, which are directly proportional to the number of viable cells in the culture [97,98]. 

**Transport assays across polarized cell monolayers:** Polarized confluent cell monolayers are created by seeding the intact cells on a permeable membrane support matrix (inserts) that fits into an assay chamber, isolating the apical compartment from the basolateral compartment. The cells will reach confluence, becoming monolayers of polarized cells. P-gp, and possibly other transporters, depending on the cell type, will be expressed on the apical membranes of epithelial cells carrying out the substrates’ transport from inside the cells into the assay buffer in the apical compartment. The transport differences between the basolateral and apical compartments, or vice versa, are then measured, giving the efflux ratio of the efflux transporter [86].

In addition to cell-based studies, membrane-based assays can also be used to evaluate P-gp’s modulation (including inhibition, induction, and activation), presenting some advantages when compared to cell-based methods, including their ability to differentiate the effects of the compounds on one specific transporter, the possibility of including them in a higher-throughput mode, and the simplicity of the experiments and their maintenance after preparation [38]. Today, there are some major membrane-based assays used in the evaluation of P-gp activity modulation:

**Membrane vesicular transport assays:** Inverted membrane vesicles are produced by mechanical homogenization, ultrasound application, or nitrogen cavitation, all resulting in cell fragments that will group together to form vesicles. Some of these will be in an “inside-out” conformation, i.e., the ATP-binding site of P-gp will be exposed to the outside of these vesicles, while the inner leaflet of the plasma membrane faces the surrounding media. After obtaining the membrane vesicles, several assays can be performed to evaluate the P-gp transport activity. The amount of substrate that has been transported into the vesicles can be measured by, for example, high- performance liquid chromatography (HPLC), liquid chromatography–mass spectrometry (LC-MS) or liquid chromatography–tandem mass spectrometry (LC-MS-MS), radiolabeling, or fluorescence and ATP assays [86].

**Photoaffinity labeling assays:** There are two major approaches when using this methodology. First is the direct detection of the substrate/modulator binding to P-gp. The photolabeling agents, connected to the efflux pump ([3H]azidopine, [125I]iodoaryl-azidoprazosin, [125I]11-azidophenyl agosterol A, [125I]iodoaryl azido-rhodamine 123), produce ultraviolet (UV) radiation for several minutes. The radioactively labeled P-gp is solubilized, separated by gel electrophoresis, visualized, and quantified by autoradiography. The second approach is based on the measurement of a radioactively labeled ATP analog, 8-azido-ATP, which, under non-hydrolytic conditions, can be followed by UV irradiation, size fractionation, and autoradiography. The formation of this analog occurs when the stabilization of the intermediate state of P-gp is achieved [38]. 

As already mentioned, the highest P-gp expression occurs in the epithelial cells of the intestine, bile ducts of the liver, proximal kidney tubules, and BBB. Therefore, it appears reasonable that the majority of studies performed in this field use BBB, liver, kidney, and intestine cell lines (Figure 4).

In the 1990s, several *in vitro* cellular models of the BBB started to be developed, providing an alternative and/or complementary approach to the *in vivo* techniques [99]. The human brain endothelial capillary cell line (hCMEC/D3 cells) is one of the most extensively characterized cell lines of the human BBB. It is commonly used for the evaluation of P-gp-mediated transport, as these cells retain the major morphological and functional characteristics of the BBB endothelial cells, even in monoculture, without glial cells. This cell line was developed through the immortalization of primary human brain capillary endothelial cells through the co-expression of the human telomerase reverse transcriptase and SV40 large T antigen, via a lentiviral vector system [100].

Other examples of *in vitro* models of the BBB include static models that use endothelial cell monocultures. Brain vascular endothelial cells, obtained from several sources, such as cows, pigs, rodents, primates, and humans, are grown in microporous semipermeable membranes within a vertical side-by-side diffusion system. This setup enables the interchange of compounds between the luminal and abluminal compartments. After reaching confluence in the luminal compartment, this model holds huge potential for performing studies on drug permeability and binding affinity. The static 2D models of the BBB can also be used, which use the co-culture of endothelial and glial cells. In these models, the inclusion of glial cells and the induction of glial–endothelial interactions will increase the expression of brain endothelial marker enzymes, transporters (like P-gp), and tight junction proteins, inducing a phenotype that more closely resembles that observed *in vivo* [99].

On the other hand, since the 1970s, isolated brain microvessels have been successfully used to provide information about the transport processes mediated by different transporters [99]. Chaves and colleagues employed this technique to evaluate the expression and activity of P-gp and breast cancer resistance protein (BCRP) in the BBB of rats upon subchronic continuous morphine infusion and naloxone-precipitated morphine withdrawal. After the administration to rats of (a) continuous morphine (intravenously (i.v.), for 120 h), (b) escalating morphine dosing (10–40 mg/kg, intraperitoneally (i.p.), for 5 days), or (c) a chronic morphine regimen (10 mg/kg, subcutaneously, for 5 days), followed by a withdrawal (2 days) and treatment (3 additional days), the isolation of microvessels from rat brain cortices was performed. Microvessels were obtained after mechanical dissection, centrifugation, and filtration through two successive nylon meshes (100 mm and 20 mm, respectively), and then both P-gp and BCRP expression/activity were analyzed by several techniques, including ribonucleic acid (RNA) extraction, quantitative real-time (qRT)-PCR, Western blotting, and protein quantification by UHPLC-MS-MS. The results showed that the continuous i.v. administration of morphine did not alter the P-gp/BCRP protein levels, while in the other groups these levels were increased. The authors concluded that a subchronic morphine administration does not induce a significant effect on P-gp/BCRP protein expression levels in the rat BBB [101].

To predict toxic responses occurring *in vivo*, several *in vitro* liver models have also been extensively studied, particularly for screening interactions mediated by different transporters and metabolic enzymes. Commonly used immortalized liver-derived cells include immortalized cell lines, like the HepG2 and HepaRG cell lines, and primary isolated hepatocytes [102]. However, while in HepG2 the expression of genes involved in phase I and II metabolism will change between passages, HepaRG cells maintain the levels of several liver-specific functions, CYP450 enzymes, nuclear receptors, membrane transporters (including P-gp), and phase II enzymes [103]. Recently, Carabias and coworkers used the human hepatocellular carcinoma (HCC), HepG2/C3A (CRL-10741, a clonal derivative of the HepG2 cell line), and PLC/PRF/5 cell lines to successfully demonstrate that galectin-1 (a β-galactoside-binding protein abundantly expressed in the tumor microenvironment, highlighted as a hallmark of hepatocellular carcinoma’s progression, aggressiveness, and metastasis given its potential involvement in chemoresistance) protects these cell lines from doxorubicin- and sorafenib-induced cell death. The researchers conducted cell viability assays to assess the cytotoxicity induced by doxorubicin (2 µM, for 48 or 72 h) and sorafenib (30 µM, for 24 h), in the presence/absence of verapamil (a P-gp inhibitor) or probenecid (an MRP2 inhibitor), for 30 min. The aim was to investigate P-gp’s involvement in this resistance phenomenon. The results indicated that a higher expression of galectin-1 in HepG2 cells reduced the intracellular accumulation of doxorubicin or sorafenib, probably due to the increase in P-gp protein expression (GAL1-overexpressing HepG2 cells exhibited increased P-gp protein levels). However, in PLC/PRF/5 cells, no P-gp protein expression was observed, nor was doxorubicin- or sorafenib-induced cytotoxicity affected by galectin-1. Therefore, the authors concluded that galectin-1 was only capable of protecting HCC cells from doxorubicin- and sorafenib-induced cell death [104].

Primary cultures of hepatocytes can also be useful for the study of transporters’ activity and expression, and for the development and identification of P-gp modulators. However, they can undergo a differentiation process that will cause changes in cell morphology, structure, polarity, gene expression, and liver-specific functions. Romiti et al. used primary cultures of rat hepatocytes to assess the capacity of curcumin to interact with P-gp, reporting that, in this cellular model, a spontaneous overexpression of the *MDR1/ABCB1* gene was observed. They performed RHO123 accumulation assays to evaluate P-gp-mediated transport, showing that curcumin inhibited the P-gp transport activity in a concentration-dependent manner. Through Western blotting analysis, they observed a decrease in P-gp expression in cells exposed to curcumin (25 µM, for 72 h). After performing the cytotoxicity assays, the authors concluded that curcumin (50 to 150 µM) was cytotoxic to the freshly plated hepatocytes in the first 24 h. However, the cells developed resistance in the presence of dexamethasone or dimethyl sulfoxide, compounds known to benefit the maintenance of differentiated cells’ functions (including the delay of spontaneous P-gp overexpression during culture), being significantly less cytotoxic when added to cells cultured for 24 or 48 h, which was reverted when exposed to verapamil [105].

For *in vitro* studies performed at the kidney level, the majority of researchers use the HK-2 (human kidney-2) cell line, an immortalized proximal tubule epithelial cell line derived from a normal human adult kidney. The cortical proximal tubule segment from a healthy kidney was isolated, cultured, and immortalized by transduction with the human papilloma virus 16 (HPV-16) *E6/E7* genes. Therefore, the HK-2 cell line preserves the phenotypic and functional characteristics of renal PTECs [106]. Nieri et al. investigated the interactions between some endogenous and synthetic cannabinoid molecules and P-gp in HK-2 cells, using calcein-AM as a P-gp substrate. The transport activity of this efflux pump was diminished by anandamide (20 µM, for 15 min), but not by 2-arachidonoylglycerol or palmitoylethanolamide (20 µM, for 15 min), since there was an accumulation of calcein in the PTECs. Furthermore, it was also suggested that the observed P-gp modulation occurred via a cannabinoid-receptor-1- and -2-independent pathway, as no mRNA coding for these receptors was detected by RT-PCR in HK-2 cells [89].

However, other cell lines, like Madin–Darby canine kidney (MDCK) cells and normal rat kidney-52E (NRK-52E) cells, have also been used for the assessment of P-gp modulation, as further described. The MDCK cell line is usually transfected with the *MDR1/ABCB1* gene in order to make it possible to carry out comparisons between the MDCK wild-type (MDCK-wt) and the *MDR1/ABCB1* gene-transfected MDCK (MDCK-MDR1) cell lines, unequivocally proving the potential involvement of P-gp in the observed drug-mediated alterations. In 2005, Iqbal and coworkers validated an HPLC method for the quantification of RHO123, with the aim of assessing the impact of different compounds on P-gp activity. This method was further applied for the evaluation of the effects of immunosuppressants on the HK-2, MDCK, and MDR1-MDCK cell lines. Additionally, the exposure of these three cell lines to RHO123 (5.0 µM) in the presence/absence of tacrolimus (0.1 µM) and sirolimus (0.1 µM), for 2 h, did not cause any alterations in P-gp transport activity [107]. Moreover, Ivanova et al. evaluated the ability of deoxynivalenol, a mycotoxin produced by the fungus *Fusarium*, to modulate P-gp transport activity. To evaluate P-gp activity, the researchers performed a calcein accumulation assay, finding that MDCK-MDR1 cells had a calcein accumulation 4.7 times lower than MDCKII-wt cells, demonstrating higher P-gp-mediated efflux in the transfected cells. By exposing MDCK-MDR1 cells to deoxynivalenol (0.04 µM–20 mM, for up to 72 h), they concluded that this compound caused a nonsignificant decrease in the accumulation of calcein-AM. The subsequent analysis of the deoxynivalenol concentration by liquid chromatography–high-resolution mass spectrometry (LC-HRMS) revealed that this compound is also a substrate of the efflux transporter. In this way, they concluded that deoxynivalenol reduced the intracellular accumulation of calcein-AM, which was also exported by P-gp [108]. 

Despite the usefulness of the previously described kidney cell lines, researchers have also investigated other *in vitro* models to study the transporters’ activity at the kidney level. Vormann et al. described a functional, easy-to-handle, decent-throughout, and user-friendly kidney-on-a-chip model that can be used to study the effects of compounds in 40 parallel-cultured renal tubules, in which it is possible to assess barrier function (including transporters’ activity), viability, lactate dehydrogenase leakage, and immunohistochemical staining. All of these proprieties were validated by several methodologies: (a) immunostaining: exposure to a nephrotoxic agent, like cisplatin, induced disruption of the epithelium, decreased cell viability, increased effluent lactate dehydrogenase (LDH) activity, and induced changes in the expression of tight junction markers; (b) fluorescence-based transporter assays: RHO123 efflux was significantly decreased in the presence of cyclosporine A [109]. Vriend et al. demonstrated a 3D microfluidic PTEC model more focused on the evaluation of drug–transporter interactions, evaluating several renal transporters (including P-gp) in a platform consisting of 96 chips. The new *in vitro* model was validated through the evaluation of the expression of genes coding for several transporters (such as organic anion transporter 1 (OAT1), organic cation transporter 2 (OCT2), P-gp, and MRP2/4), which presented similar levels to those expressed in 2D static cultures. Additionally, P-gp and MRP2/4 function was assessed by the calcein-AM assay, in the presence of specific inhibitors (PSC833, and MK571 and KO143, respectively), where increased accumulation of calcein was observed in the presence of such inhibitors. Furthermore, this model enabled medium-/high-throughput screenings, being compatible with many high-content imaging platforms [110]. 

For the investigation of DDIs at the intestinal P-gp level, the most used *in vitro* model of human intestinal epithelial cells is the Caco-2 cell line, which was originally developed from a human colon adenocarcinoma [111]. The Caco-2 cell line holds most of the morphological and functional properties of human enterocytes, including the typical brush border with microvilli and tight junctions, as well as uptake/efflux transporters. Ezuruike and coworkers assessed the possible interactions between extracts of 27 popular Nigerian “antidiabetic” plants and glibenclamide, a conventional antidiabetic drug that is a P-gp substrate, with respect to potential interferences in P-gp transport activity. RHO123 efflux assays were performed in vincristine-resistant Caco-2 cells, where eight extracts were selected for the next assay due to their P-gp-inhibitory capacity. Using Caco-2 monolayers, the researchers reduced their list to three extracts with similar capacity to verapamil for reducing the efflux ratio of glibenclamide: *Syzygium guineense*, *Terminalia avicennioides*, and *X. American* (100 µg/mL, for 2 h) [112]. Our group has used this cell line in several *in vitro* studies focused on the evaluation of the ability of different compounds to induce and/or activate P-gp [21,83,90,91,94,113,114], and which will be described in more detail in the following sections. 

Despite the great usefulness of the Caco-2 cell line, there are other cell lines that can also be used in the evaluation of P-gp’s expression and activity at the intestinal level. Silva and coworkers tested the SW480 cell line to verify its applicability in the screening/identification of P-gp inducers/activators, along with the investigation of the potential effects of six oxygenated xanthones (OXs) on P-gp expression/activity and, subsequently, on the cytotoxicity induced by mitoxantrone, a known P-gp substrate. The authors observed the following: (a) after 24 h of exposure, OX2, OX4, OX5, and OX6 (20 µM) significantly increased P-gp expression, demonstrating their induction effect; (b) after 90 min of exposure, OX1, OX2, OX4, OX5, and OX6 (20 µM) significantly increased P-gp activity by rapidly eliminating the RHO123 from the cells, proving their role as P-gp activators. However, the tested OXs were not capable of protecting both SW480 and Caco-2 cells from the cytotoxicity induced by mitoxantrone, with this lack of protection explained by the different binding locations of OXs and mitoxantrone within the P-gp drug-binding pocket, as observed *in silico* by docking simulations performed in a human P-gp model. Interestingly, for the first time, the SW480 cell line was considered to be a suitable *in vitro* model for the assessment of drug-mediated P-gp modulation [113].

Some researchers developed another intestinal *in vitro* model derived from the intestinal porcine epithelial cells-2 (IPEC-2), which were transfected with a plasmid containing the human *MDR1/ABCB1* gene sequence. The resulting cell line, the iP-gp cell line, demonstrated an overexpression of human P-gp and a low expression of porcine P-gp (and BCRP), as well as high paracellular tightness, proving its value for the assessment of potential drug interactions with P-gp [115,116]. The researchers successfully tested the transepithelial efflux of RHO123 in the iP-gp and MDCK-MDR1 cell lines, obtaining the highest efflux ratios in the iP-gp cells, probably due to a tighter paracellular pathway. These effects were inverted in the presence of zosuquidar, a known P-gp inhibitor, proving the involvement of P-gp in the RHO123 transport [116].

Although some researchers choose to focus their study of DDIs on a specific organ, we must be aware and remember to correlate them with an entire, functional body. As such, some researchers choose to analyze and compare the overall results obtained in cell lines representative of different organs. In 2007, van de Water and coworkers simultaneously evaluated P-gp expression (by reverse-transcription-PCR and Western blotting) and activity (with the calcein-AM assay) in three rat epithelial cell lines: intestinal IEC-6 cells, and kidney GERP and NRK-52E cells, in order to characterize new *in vitro* models suitable for drug discovery. They concluded that both cell lines expressed functional P-gp, as well as MRP [117]. Another group of researchers investigated the transport of nanoparticles (cadmium quantum dots) in liver (HepG2) and kidney (HK-2 and MDCK) cells. They proved that the HepG2 and HK-2 cell lines are suitable for the assessment of P-gp-mediated DDIs, since a significant concentration- and time-dependent induction of ABC transporters, including P-gp, was observed upon exposure to free Cd^2+^ [118]. 

*In vitro* assays used in the assessment of drug-mediated P-gp modulation are used not only for DDI screening, but also for the evaluation of specific effects on P-gp’s activity and/or expression. Although it is possible to quickly obtain a remarkable amount of results with *in vitro* methods, contrary to what is possible with the *in vivo* methods, the *in vitro* assays do not fully represent the properties of a real functioning body. Additionally, since several cell lines are derived from the laboratory animals themselves, extrapolation of the results to human beings becomes even more complicated [119]. 

Therefore, as described above, *in vitro* methods are an important laboratory tool for the evaluation of a large number of compounds (at varying concentrations and with different exposure times) that allow for the determination of DDIs that specifically involve changes in P-gp expression and/or activity. Nevertheless, these do not completely mimic the human body and must be complemented with other types of techniques, such as *ex vivo* and *in vivo* models.

### 3.3. Ex Vivo Models

An alternative approach to explore the transporters’ expression and/or activity involves the use of *ex vivo* techniques. In this methodology, after removing the organ from the living body, it is placed in chambers that mimic the physiological conditions of the animal, including nutrient and oxygen levels, as well as organ or tissue viability. For example, to explore P-gp’s function in intestinal epithelial cells, an *ex vivo* method was developed using the everted intestinal sacs of rats. After preparation, these sacs were filled with a specific fluorescent P-gp substrate (RHO123) and maintained in aerated buffer-containing chambers at 37 °C, in the presence/absence of the potential modulator. The posterior analysis was simply performed on the samples of mucosal medium by measuring RHO123 fluorescence by spectrofluorometry [120,121]. 

Martins and coworkers studied the effects of OX1-6 on P-gp’s induction and/or activation. The authors successfully proved the protection of Caco-2 cells against paraquat (PQ)-induced cytotoxicity in the presence of OX1-*6 in vitro* (20.0 µM, for 24 h), while also demonstrating the P-gp induction and/or activation effects of these derivatives. The most promising results were obtained for OX6, which was selected for the *ex vivo* assays. Adult Wistar Han rats were anesthetized, and the distal portion of the ileum (20 cm) was removed, washed, and immediately used to prepare the rats’ everted intestinal sacs. The intestinal portions were placed in a chamber in the presence/absence of OX6 (20 µM), with and without zosuquidar (10 µM), a known P-gp inhibitor, and 10 min later the RHO123 (300 µM) was added. The RHO123 concentration was determined by spectrofluorometry in samples collected from the mucosal medium, every 5 min, for a 45 min period. The *ex vivo* results indicated a significant increase in P-gp activity in the presence of OX6, confirmed by the suppression of this effect in the presence of zosuquidar [21]. 

Also using rats’ everted intestinal sacs, Rocha-Pereira et al. evaluated the potential of the thioxanthonic derivative 1-(propan-2-ylamino)-4-propoxy-9H-thioxanthen-9-one (thioxanthone 5, TX5) to affect P-gp activity *ex vivo*. The RHO123 efflux was significantly increased in the everted intestinal sacs of the distal ileum in the presence of TX5 (20 µM), an effect abolished by the P-gp inhibitor verapamil, thus demonstrating TX5’s ability to activate P-gp. However, the TX5-mediated increase in P-gp activity detected in the everted sacs of the distal ileum after direct contact with TX5 was not observed in everted intestinal sacs obtained from the proximal ileum. However, despite the promising *ex vivo* results, when TX5 hydrochloride (10 mg/kg b.w.) was administered to adult male Wistar Han rats, no significant differences in P-gp expression levels were detected between brush border membranes isolated from the distal portions of the intestines of control and TX5-treated rats 24 h after TX5 administration. In agreement with this, the P-gp activity also remained unaffected in everted intestinal sacs of the distal ileum from TX5-treated rats. Lastly, the authors aimed to assess whether the observed TX5-mediated P-gp activation (detected *ex vivo* in the distal portion of the ileum) could impact the bioavailability of P-gp substrates upon the intraduodenal administration of a TX5 bolus *in vivo*. However, the *in vivo* results showed no alterations in the digoxin plasma levels between control and TX5-treated rats [122].

In another study, it was demonstrated that human precision-cut intestinal slices were an adequate *ex vivo* model to study P-gp activity and the respective transporter-mediated DDIs. The human intestinal tissue was obtained from different patients during surgery, rapidly transported to the laboratory, and washed, and the blood and luminal contents were removed. The mucosal layer was cut into slices, which were individually incubated in a prewarmed chamber (37 °C, 95% O_2_, and 5% CO_2_). Slices of human duodenum, jejunum, ileum, and colon were incubated with/without the following P-gp inhibitors: verapamil (0–50 µM), cyclosporine A (0–20 µM), quinidine (0–200 µM), ketoconazole (0–50 µM), PSC833 (0–2 µM), and CP100356 (0–5 µM), for 3 h, in the presence/absence of RHO123 (up to 10 µM). The results obtained from the RHO123 accumulation assays demonstrated that all of the compounds were capable of inhibiting P-gp, demonstrating the usefulness of this *ex vivo* model for the assessment of compound-mediated P-gp inhibition. In terms of the comparison between the different intestinal regions, the ileum emerged as the intestinal region with the most pronounced enrichment in P-gp, while there were no significant differences observed among the other intestinal regions [119].

In this way, it is deducible that *ex vivo* techniques are very useful for the evaluation of drug-mediated P-gp modulation, since they allow for the direct evaluation of the immediate effects of compounds on P-gp’s expression and/or activity at the organ/tissue level.

### 3.4. In Vivo Models

Despite the importance of *in vitro* and *ex vivo* assays, there is a need to develop adequate *in vivo* models to evaluate the impact of transporters in drug pharmaco-/toxicokinetics. In these types of models, animals (such as mice) are used to evaluate the effects of P-gp modulation on the disposition of the xenobiotics under study [85]. Nevertheless, they should be carefully planned and approved in agreement with the national or institutional guidelines for the care and use of animals [123]. Frequently, transporter-gene-knockout models are used to define the transporters’ roles in drug disposition. Humanized transporter animal models are usually used to overcome the differences between species in transporter expression and substrate specificity [85]. Although most studies include the euthanasia of the animals, several techniques have been developed to measure the desired parameters without this cost. Recently, Hernandez-Lozano and coworkers suggested the use of non-invasive nuclear imaging techniques (e.g., positron emission tomography (PET) or single-photon emission computed tomography (SPECT)) since they allow the quantification of the tissue concentrations of radiolabeled compounds. Using PET imaging of rat lungs, they successfully demonstrated that a reduction in P-gp activity affected the pulmonary disposition of inhaled P-gp substrates, consequently causing a decline in therapeutic efficacy [124]. 

Focusing again on the sites of greater P-gp expression, like the BBB, liver, kidneys, and/or intestines, it is possible to also find in the literature several studies performed *in vivo* to assess the drug-mediated P-gp modulation. De Bruyne et al. efficiently established a methodology to evaluate the efficacy of new potential P-gp modulators in the brains of male wild-type and *mdr1a* (−/−) mice (also known as P-gp-knockout mice), classifying [123I]-4-(2-(bis(4-fluorophenyl)methoxy) ethyl)-1-(4-iodobenzyl)piperidine) as a SPECT tracer for imaging P-gp at the BBB [125]. Ceré and coworkers used adult female Wistar rats (in particular, lactating postpartum and ovariectomized rats) to examine the hepatic P-gp modulation induced by prolactin, along with the ways in which it modifies the export of xenobiotics from the liver to the bile. After exposure to prolactin (300 µg/day, for 7 days, via osmotic minipumps), P-gp’s expression and activity were assessed by Western blotting and RHO123 accumulation assay, respectively. The researchers founded that P-gp’s expression and activity were increased in the presence of prolactin, suggesting a higher elimination of xenobiotics in hyperprolactinemic circumstances, including those with therapeutic purposes [126]. 

More recently, Takeda et al. evaluated the modulation of the expression and function of intestinal efflux transporters, including P-gp, in cisplatin-induced acute-kidney-injured rats. Three days after exposing male Sprague Dawley rats to cisplatin (5 mg/kg, i.p.), the authors used a conventional loop technique to assess the expression and activity of P-gp and BCRP (using 6α-methylprednisolone, RHO123, and gatifloxacin as substrates). They found that P-gp expression significantly decreased in the ileum of rats’ small intestines in the case of cisplatin-induced acute kidney injury rats (to 38% compared with the normal rats). Therefore, the researchers concluded that P-gp could maintain its role by increasing its activity. However, no changes were observed in the BCRP expression in the cisplatin-induced acute kidney injury rats [127]. 

Most researchers choose to perform their studies not only exclusively *in vivo*, but also in conjunction with *in vitro* and *ex vivo* techniques. To evaluate the *mdr1*/*abcb1* gene expression patterns associated with cisplatin-induced nephrotoxicity, male Sprague Dawley VAF1 albino rats were exposed to cisplatin (0.5 mg/kg/day or 1 mg/kg/day, for 24 h or 7 days), euthanatized, and their livers and kidneys removed for macroscopic and histopathological examinations and posterior microarray analyses. Along with the *in vivo* methodology, NRK52E cells and normal rat hepatocyte clone-9 cells were also treated with cisplatin (0–5 µg/mL, for 24 h), and P-gp expression was examined by RT-PCR. In the kidneys of animals that received cisplatin (0.5 or 1 mg/kg/day, for 7 days), the results showed a renal proximal tubular necrosis and the upregulation of several genes, including *mdr1/abcb1* and genes coding for tissue remodeling proteins (e.g., clusterin1, IGFBP-11, and TIMP-11), indicating cisplatin resistance and also necrosis with tissue regeneration. *In vitro* experiments suggested that liver cells were more sensitive to cisplatin than kidney cells, which did not occur in the *in vivo* experiments. The authors urged caution with regard to the extrapolation of data obtained from *in vitro* experiments to *in vivo* systems, since this *in vitro* system was not predictive of the target organ of cisplatin-mediated toxicity [87]. Ahmed et al. aimed to study the effects of lyophilized freshly prepared grapefruit juice on P-gp exportation, *in vitro* and *in vivo*, in human uterine sarcoma MES-SA/DX5v cells and in white New Zealand male rabbits, using doxorubicin and timolol maleate as substrates, respectively. *In vitro* studies showed an accumulation of doxorubicin in MES-SA/DX5v cells (18.3% compared to the control cells) upon exposure to doxorubicin (1 µM), in the presence or absence of lyophilized freshly prepared juice (0.05, 0.5, 5, or 50% *v/v*), for 4 h. The *in vivo* studies presented higher absorption of timolol maleate in the presence of the potential modulator, lyophilized freshly prepared grapefruit juice. It was also possible to verify that the lyophilized freshly prepared juice caused a higher inhibitory effect (70%) than commercially available juices (43%). Therefore, the authors concluded that the lyophilized freshly prepared juice had a P-gp-inhibitory effect, augmenting the absorption of timolol [128]. 

In another study, researchers studied the influence of phospholipids naturally surrounding P-gp on its transport activity. To accomplish that, two synthetic phospholipids, 1,2-dioctanoyl-sn-glycero-3-phosphocholineand and 1,2-didecanoyl-sn-glycero-3-phosphocholine, were studied and compared to Tween^®^ 80 and Cremophor^®^ EL (surfactants with known inhibitory effects on P-gp). The studies were performed *in vitro*, through the RHO123 accumulation/transport assay across Caco-2 cell monolayers, and *in vivo* after the administration of ritonavir (20 mg/kg, intragastric), a known P-gp substrate, followed by its extraction and quantification in the plasma of male Wistar rats (every 30 min for 150 min) using solid-phase extraction and chromatography with ultra-performance liquid chromatography–tandem mass spectrometry (UPLC-MS-MS), respectively. The authors described that the phospholipids inhibited P-gp activity in Caco-2 cells and significantly increased the intracellular accumulation of RHO123 *in vitro*, while the *in vivo* results demonstrated a significant increase in the area under the ritonavir plasma level curve within 150 min (more than 10-fold), albeit inferior to the effects observed for Tween^®^ 80, which possessed superior solubilizing effects. Therefore, it was concluded that these synthetic phospholipids demonstrated great capacity for P-gp modulation, with a high permeabilization potential [129]. 

Ballent et al. evaluated the effects of P-gp on the pharmacokinetics of ivermectin in the plasma, liver, and gastrointestinal tissues, merging the *ex vivo* and *in vivo* methodologies. *Ex vivo*, the researchers used the everted ileal sacs methodology, filled with ivermectin (3 µM), where the results indicated that ivermectin levels were higher in the presence of itraconazole (10 µM) or PSC833 (10 µM), known P-gp inhibitors. The *in vivo* methodology integrated the co-administration of ivermectin (200 µg/kg, subcutaneously) and itraconazole (5 mg, two doses, subcutaneously) or PSC833 (8.6 mg, two doses, subcutaneously) to male Wistar rats, as well as the posterior quantification of ivermectin levels using HPLC. Accordingly, the *in vivo* results showed that itraconazole/PSC833 caused a higher concentration of ivermectin in plasma and gastrointestinal tissues, proving the enhanced absorption of ivermectin [130]. A similar approach was used by Srirangam and Sagar to assess potential changes in the intestinal secretion of glibenclamide (3.6 mg/kg, at 1, 2, 4, 6, and 8 h) in the presence of a P-gp modulator, carbamazepine (90 mg/kg), using ileal sacs and albino rats (either sex). Not only did the *ex vivo* results show a lower accumulation of glibenclamide in the intestinal walls of everted sacs in the presence of carbamazepine, the *in vivo* experiments also demonstrated an increase in the absorption of glibenclamide in the gastrointestinal tract in the presence of carbamazepine, proving the relevance of P-gp in drug absorption [131]. 

In another study, an *in vitro*, *ex vivo*, and *in vivo* approach was applied to evaluate the P-gp modulation caused by *Coptidis Rhizoma* (CR, containing alkaloids like berberine, coptisine, and palmatine), using cyclosporine as a P-gp substrate. *In vivo*, male Sprague Dawley rats were orally administered with cyclosporine (2.5 mg/1.0 mL/kg) and CR (1.0 g/2.0 mL/kg), and blood samples were collected at 20, 40, 60, 180, 300, and 540 min and further evaluated for the quantification of cyclosporine levels through a specific monoclonal fluorescence polarization immunoassay. The *in vivo* results indicated that CR significantly decreased the cyclosporine levels in the blood samples. In the *in vitro* evaluations, the LS 180 cell line (human colon adenocarcinoma) was used to perform the RHO123 accumulation assays, which demonstrated that CR (100 and 200 µg/mL, for 72 h), berberine, coptisine, and palmatine (2.5, 5.0, or 10 µM, for 4 h) caused an increased P-gp-mediated efflux of RHO123. In the *ex vivo* studies, Vivid^®^ CYP450 screening kits were applied to assess the effects of CR metabolites on the activity of CYP3A, and the obtained results indicated an increase in CYP3A activity when compared to the blank. Accordingly, it was confirmed that CR enhanced the activity of P-gp and CYP3A4, further suggesting that the two have an overlap in their substrates [132].

Another type of methodology that can be used is *in situ* methods, in which the cell, for example, is evaluated within the whole organ/body. These types of assays are different from the *in vitro* models, since they are performed within the functional organ/body, and from the *ex vivo*/*in vivo* models, as there is no euthanasia of the donor. 

Using *ex vivo*, *in situ*, and *in vivo* methodologies, Bedada and coworkers studied the effects of capsaicin (present in chili peppers) on the intestinal absorption and bioavailability of fexofenadine, a P-gp substrate, in male Wistar rats. In all of the experiments, the fexofenadine concentration was determined by HPLC. In the *ex vivo* methodology, non-everted gut sacs were used. Fexofenadine alone (control group), capsaicin pretreatment (test group; 3 mg/kg, orally, for 7 days), and verapamil pretreatment (positive control group; 25 mg/kg, orally, for 7 days) were administrated to the rats, which were euthanatized on the 8th day. After the intestines’ removal and the development of the intestinal sacs, these were filled with fexofenadine (500 µg/mL), placed in a chamber, and sampled every 15 min for 2 h. The results indicated an increase in fexofenadine transport from the mucosal to the serosal surface in the ileum of capsaicin-pretreated rats, similar to what was found for verapamil (positive control). In the *in situ* single-pass intestinal perfusion assay, although the rats received the same pretreatment, on the 8th day an ileal segment of approximately 8–12 cm was surgically removed, which was then perfused with phosphate-buffered saline (pH 7.4) containing fexofenadine (50 µM), propranolol (100 µM), and phenol red (50 mg/mL), and samples were collected every 10 min for 90 min. The k_a_ values obtained after fexofenadine quantification indicated an increase in the presence of the potential inhibitor capsaicin (and also of verapamil). *In vivo*, the rats received the same pretreatment with posterior oral administration of fexofenadine (10 mg/kg) to the control and test groups, and blood samples were collected at 0.25, 0.5, 0.75, 1, 1.5, 2, 4, 8, 12, and 24 h. When compared to the control group, the bioavailability of fexofenadine in the other groups was significantly affected, demonstrating the inhibitory effect of capsaicin according to the other results and, consequently, the need for attention to potential DDIs [133].

Although the *in vivo* techniques did not permit the screening of several compounds (neither a wide range of doses/concentrations nor different exposure times), these represent a valuable tool to evaluate DDIs involving changes in P-gp transport activity, especially if the compounds under study are selected from previous *in silico* and/or *in vitro* studies. Despite their enormous usefulness, their extrapolation to the human body can be tricky, which can be bypassed through clinical trials.

### 3.5. Clinical Trials

**Figure 5:**
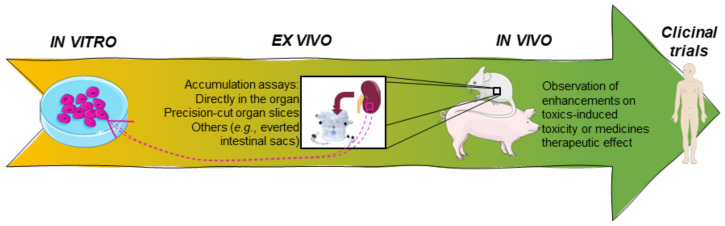
Representative scheme of the major models used in the evaluation of DDIs before moving forward to clinical trials, particularly the *in vivo* and *ex vivo* models.

After the experimentation *in vivo* using animals, the most relevant DDIs should be further demonstrated in the human body through the development of clinical trials (Figure 5). Clinical trials consist in experimentation in one or more human subjects, who are prospectively assigned to interventions, where the subsequent effects are evaluated in the human subject, expecting health-related biomedical/behavioral outcomes [134]. 

Rengelshausen and coworkers developed a randomized, placebo-controlled, double-blind crossover study in 12 healthy men, with the aim of assessing the interactions between digoxin and clarithromycin (250 mg, orally), as well as the consequences resulting from the potential interactions. Digoxin (0.75 mg) was orally administrated with a placebo or clarithromycin (250 mg) twice a day for 3 days, and the collected samples of blood and urine were processed for the quantification of digoxin in plasma and urine, using a highly sensitive radioimmunoassay. It was observed that the oral co-administration of clarithromycin increased the digoxin area under the curve by 1.7-fold when compared with the placebo. They concluded that clarithromycin, probably due to inhibition of P-gp in the intestines and kidneys, decreased the non-glomerular renal clearance of digoxin and increased its oral bioavailability [135].

Therefore, clinical trials are the final approach to perform in the drug discovery field, where the modulatory effects of the selected compound (previously selected from all of the other techniques and types of studies) are tested in the whole human body.

## 4. Drug-Mediated P-gp Modulation

**Figure 6:**
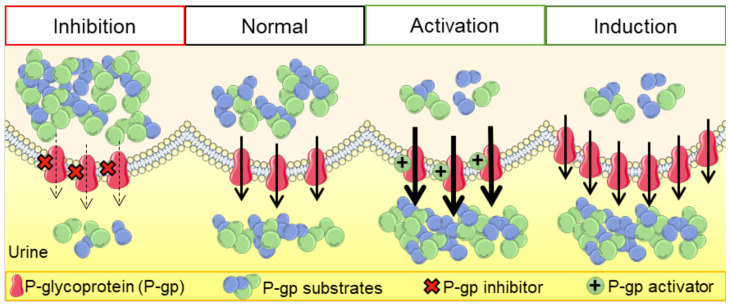
Representative scheme of the proximal tubular epithelial cells with normal, inhibited, or induced/activated P-glycoprotein.

P-gp is involved in several types of interactions, including drug–drug, food–drug, chemical–drug, herb–drug, and herb–herb interactions [136]. However, most researchers commonly refer to all of these types of interactions as DDIs. DDIs can impact the therapeutic effectiveness of drugs or even lead to the development of adverse reactions by modulating the activity of specific transporters, consequently influencing the tissue distribution of several drugs [137]. In other words, DDIs involving the inhibition or induction/activation of a given transporter may lead to an increase or decrease in the levels of the substrate, respectively [1,2]. In this way, researchers have been taking advantage of DDIs to modulate the transport activity of these transporters, aiming to modify the pharmacokinetics and pharmacodynamics of co-administered compounds [138,139]. P-gp, in the presence of inhibitors, will not effectively perform its transport function, leading to the intracellular accumulation of compounds, which can be therapeutically useful to overcome the MDR phenomenon. However, in the presence of inducers or activators, particularly at the kidney and intestinal levels, P-gp can facilitate the export of compounds, resulting in their increased/accelerated excretion. This phenomenon can be a potential antidotal strategy in scenarios involving intoxication and drug-induced nephrotoxicity, as it aids in the removal of toxic substances from the body at a faster rate (Figure 6).

Despite all of the research conducted in this field, there are some considerations that need to be addressed when evaluating the modulation of transporters’ expression and/or activity. Especially in clinical practice, it is very common to simultaneously administer several medications to elderly patients with impaired cardiac, liver, and/or renal function. However, during drug development studies, young and healthy volunteers are normally involved. Thus, it is important to address the extent to which the data obtained in these studies will be extrapolated and clinically relevant, as well as whether the existing databases will be enough to know the major DDIs among the currently prescribed drugs [14].

From a scientific research perspective, there are also several limitations to consider when interpreting the results in the evaluation of DDIs. First, the interaction of a substrate with other transporters needs to be taken into account, as most xenobiotics are transported by more than one transporter, including both uptake and efflux transporters, which means they may not be specific substrates of a single transporter [14]. In addition, it is important to consider that several substrates of transporters, including P-gp, are also metabolized by one or multiple phase I and/or phase II enzymes, such as CYP450 enzymes, and this interplay between transporters and metabolizing enzymes can result in complex pharmacokinetic profiles during multidrug interactions [4,14]. As a result, along with the evaluation of the activity of modulators, it is crucial to evaluate the proportion of a compound transported by P-gp, as well as the extent to which it is metabolized and transported by other enzymes and transporters. This comprehensive assessment is remarkably needed when considering the activity of modulators in DDIs to avoid potential toxic effects or a lack of therapeutic effect [3]. Second, modulators (whether these are inhibitors, inducers, or activators) may not be specific to a single transporter and, as such, can alter the transport activity of more than one transporter simultaneously (or even that of the drug-metabolizing enzymes) [14]. Third, a xenobiotic can be a substrate of a particular transporter and may also behave as inhibitor [3]. Interestingly, P-gp’s expression and activity are frequently altered by its own substrates, which leads to consequent changes in their pharmacokinetics, bioavailability, toxicity, and therapeutic response, leading to DDIs among P-gp substrates [140]. On the other hand, there is also the possibility of dual effects over time, i.e., some modulators can cause different interactions over time, acting as an inhibitor and then, after some time, as an inducer [3]. Fourth, certain disease situations can affect the expression of transporters, as well as their respective functions, which, specifically at the kidney level, can lead to increased accumulation of xenobiotics and waste products and, consequently, progress to kidney failure [4,141]. 

Lastly, there are several specific characteristics of compounds that should be considered: (i) The selection of compound doses should be made with caution, as dose-dependent DDI’s can occur for certain efflux pumps (especially P-gp). However, it is important to note that protein saturation can suddenly alter the expected effects. (ii) The therapeutic index of the substrate can be narrow, meaning that even a small difference in dose may lead to therapeutic failure or, worse, to toxicity. (iii) The potency of a modulator (whether it is an inhibitor, inducer, and/or activator) should be sufficient to effectively change the substrate concentration to the desired substrate level in specific tissues. This becomes particularly important when administering more than one xenobiotic simultaneously. (iv) The intracellular concentration of highly permeable drugs can be significantly altered if they are also absorbed by passive transport (the transport is not exclusively dependent on P-gp activity and on its respective modulation). (v) The route of administration can have distinct outcomes on the (pharmaco)toxicokinetics of xenobiotics [3]. 

The first studies conducted on P-gp were primarily motivated by its impacting on drug resistance to anticancer agents. The primary focus of these studies was to develop effective strategies for remarkable P-gp inhibition, with the aim of overcoming drug resistance and improving the therapeutic efficacy of anticancer drugs. In other words, the objective was to inhibit the export of anticancer agents from tumor cells, increasing/prolonging their therapeutic effect. However, the presence of P-gp in other cells of the human body, other than tumor cells, makes this inhibition strategy problematic because chemical inhibitors do not discriminate P-gp expressed in normal tissues of the body from that found in tumor tissues, leading to the accumulation of several toxic xenobiotics and endobiotics in different tissues of the human body [19,142]. However, from these studies, it was possible to identify several compounds as P-gp inhibitors (Table 2). The mechanism of P-gp inhibition is thought to involve one of four pathways: direct inhibition of P-gp-binding sites that block the transport of substrates (most frequent), ATP-binding inhibition, obstructing ATP hydrolysis, and affecting the linkage between ATP hydrolysis and substrate translocation [143]. 

More recent studies have focused on the induction/activation of this efflux pump to avoid the toxicity caused by some harmful P-gp substrates, being proposed as a potential antidotal pathway. That is, the increase in P-gp expression/activity may be highlighted as a tissue protection strategy, limiting the intracellular accumulation of its substrates and, consequently, reducing their toxicity. P-gp inducers augment the expression of the *MDR1/ABCB1* gene by interacting with transcription factors, leading to higher protein expression and, consequently, a higher number of transporters exporting compounds on the tissues’ apical barriers. On the other hand, P-gp activators have the benefit of immediately increasing P-gp activity without interfering with the P-gp protein expression, being a faster process when compared with the use of P-gp inducers [21,113,142]. Table 2 lists the enormous variety of known P-gp substrates and modulators, including inhibitors, inducers, and activators.

### 4.1. Modulation of P-gp at the Intestinal Level

All of the food and drink ingested by humans are digested, and the resulting nutrients and water are absorbed within the gastrointestinal tract. Furthermore, many of the existing drugs are administered orally, being absorbed primarily in the gastrointestinal tract, with a particular emphasis in the intestines. In this way, epithelial cells are frequently exposed to distinct xenobiotics, many of them toxic, which should be efficiently excreted and metabolized by efflux transporters and metabolic enzymes, respectively. Therefore, enterocytes form a barrier with two functions: defense and detoxification. Relative to the intestinal transporters, it is noteworthy that influx transporters play a specific and important role in the absorption of orally administrated xenobiotics, conditioning their bioavailability and therapeutic efficacy. On the other hand, efflux transporters should be used therapeutically in order to decrease the xenobiotics’ toxicity, including intoxication situations [10].

Between efflux transporters, P-gp regulates the molecules’ transport from the enterocytes into the intestinal lumen, playing an important role in their oral bioavailability and clearance [10,149]. On the other hand, it is essential to acknowledge that P-gp modulation can also be a contributing factor to DDIs involving substances that are substrates of this efflux pump [10]. The well-studied drug-mediated interactions involving intestinal P-gp can be taken advantage of in two major ways: co-administration of P-gp inhibitors, to augment its substrates’ absorption and bioavailability (potentially substrates with important therapeutic effects), or co-administration of P-gp inducers/activators, with the goal of efficient/fast elimination of toxic substrates. Additionally, beyond the previously mentioned therapeutic potential of specific DDIs, it is crucial to recognize that DDIs can also occur inadvertently when multiple drugs are administered concurrently.

Over the years, several studies have explored the capacity of distinct compounds to modulate P-gp expression and/or activity at the intestinal level, as well as the impact of such modulation on the accumulation or bioavailability of P-gp substrates. For example, Taubert et al. evaluated P-gp-mediated transport of clopidogrel (1 µM) by performing efflux experiments in Caco-2 cells, in the presence/absence of several inhibitors (omeprazole (6–60 µM), cyclosporin (2–20 µM), verapamil (25–250 µM), quinidine (20–200 µM), and elacridar (0.12–1.20 µM), for 20–120 min). The researchers observed a 2.5-fold increase in the intracellular accumulation of clopidogrel, achieving higher bioavailability [150]. In another study, the inhibitory effect of polyethylene glycols on P-gp activity was examined through RHO123 accumulation/efflux assays across rat intestinal membranes in an *in vitro* diffusion chamber system. The RHO123 efflux was reduced in the presence of polyethylene glycols (range of concentrations: 0.1–20%, *v*/*v* or *w*/*v*, over 0–120 min) or their derivatives (monolaurate, monooleate, or monostearate), in a concentration-dependent manner. The intestinal absorption of RHO123 was also superior *in situ* in the presence of polyethylene glycol (20%, *v*/*v*), as evaluated in a closed-loop method. This methodology indicated a higher accumulation of RHO123, once again suggesting the inhibitory effect of the polyethylene glycols and their derivatives [151]. The inhibitory effects of some curcuminoids, extracted from *Curcuma longa* and *Curcuma* sp., were assessed *in vitro* through RHO123 accumulation assays in Caco-2 cells. Curcumin, demethoxycurcumin, and bisdemethoxycurcumin (25–50 µg/mL, for 30 min) increased the apical-to-basolateral transport, while curcumin and demethoxycurcumin decreased the basolateral-to-apical transport. The authors concluded that both plant extracts, *Curcuma longa* and *Curcuma* sp., were capable of increasing the intracellular accumulation of RHO123 [152]. More recently, Costa et al. studied the influence of curcumin on P-gp activity under static and dynamic conditions. In order to carry out a natural vs. synthetic comparison, the researchers exposed Caco-2 cells to curcumin or an arylmethyloxy-phenyl derivative (0–90 µM, for 0–48 h) and evaluated the accumulation/efflux of RHO123 in two systems: bioreactors, and transwells. The researchers concluded that no significant differences between the natural and synthetic compounds were observed. However, P-gp activity was reduced by both compounds and, additionally, to even lower levels in the presence of flow (average inhibition values of 98% and 100% in static and dynamic conditions, respectively) [153].

On the other hand, P-gp induction/activation could provide a novel therapeutic approach in the treatment of several diseases, or in intoxication scenarios, and has also already been explored in several studies. For example, several 1,4-naphthoquinone derivatives (1–8) were assessed for their potential as P-gp modulators, as well as for the compensation of intestinal inflammation. The researchers exposed Caco-2 cells to lipopolysaccharide, a known inducer of intestinal inflammation (1 µg/mL, for 4 days) and P-gp inhibitor, and 1,4-naphthoquinone derivatives 2, 3, and 4 (20 µM, for 24 h), and the P-gp and *MDR1/ABCB1* gene expression was analyzed by Western blotting and qPCR, respectively. The results demonstrated suppression of the lipopolysaccharide-induced alterations. Additionally, Caco-2 cells were exposed to derivatives 1–8 or rifampicin (20 µM, for 24 h), used as a positive control, and then RHO123 accumulation/efflux assays were conducted by flow cytometry. All of the derivatives reduced the intracellular accumulation of RHO123 in Caco-2 cells, except for 1,4-naphthoquinone derivative 1, proving their role in the induction of P-gp. Derivative 7 had the highest effect on P-gp transport activity, achieving 39% RHO123 accumulation when compared to the control. However, in this study, it was not demonstrated whether these effects on P-gp activity impacted the toxicity of the P-gp substrates [154].

Nevertheless, our group has published several studies carried out at the intestinal level, aiming to propose the positive modulation of P-gp as a therapeutic strategy. For example, at the intestinal level, studies showed that P-gp inducers like doxorubicin can significantly increase the P-gp expression/activity in Caco-2 cells, leading to a more efficient transport of toxic substrates like PQ to the extracellular matrix, resulting in decreased intracellular accumulation of PQ and, consequently, in a reduction in the cytotoxicity of the herbicide. Furthermore, TXs, compounds that hold a dibenzo-γ-pyrone tricyclic scaffold, and xanthone derivatives have also been shown to be capable of inducing and/or activating P-gp, preventing the cytotoxicity induced by their toxic substrates [21,28,83,91,113,122,142,155]. Indeed, Silva et al. demonstrated a way to decrease the toxicity of PQ using dihydroxylated xanthones (Xs) or TXs as P-gp inducers and/or activators [83,91]. Furthermore, one of these thioxanthonic derivatives, TX5, was also tested *ex vivo* and *in vivo*, demonstrating increased RHO123 efflux in the presence of TX5 (20 µM) using rats’ everted intestinal sacs, which indicates its P-gp induction/activation effect [122]. Lopes et al. also successively characterized eight distinct thioxanthonic derivatives, particularly chiral aminated thioxanthones (ATXs), as P-gp activators [142]. Moreover, one of the tested compoundsATX 2 (−), significantly increased the P-gp expression in Caco-2 cells after 24 h of incubation, modulating P-gp expression differently from its enantiomer, ATX1 (+), thus suggesting the existence of enantioselectivity in the induction mechanism. More recently, OXs were identified as potential P-gp inducers and activators at the intestinal level, using Caco-2 cells and SW480 cells as *in vitro* models, along with everted intestinal sacs from adult Wistar Han rats as an *ex vivo* model. In both studies, the *in vitro* studies showed a significant increase in P-gp expression and/or activity in the presence of all OXs except for OX3, proving their potential as P-gp inducers and/or activators. OX6, the most promising xanthonic derivative, was then selected for the *ex vivo* studies, in which augmented P-gp transport activity was observed [21,113]. However, different thioxanthonic derivatives were also already reported as P-gp inhibitors [79,144]. In 2012, Palmeira and coworkers assessed the inhibitory effects of ATXs and their effects on antineoplastic agents’ cytotoxicity. The authors exposed K562 (human chronic myelogenous leukemia) and K562Dox (derived from K562 by doxorubicin-stimulated overexpression of P-gp) cell lines to test compounds (10 or 20 µM, for 1 h) along with RHO123 (1 µM), the fluorescence of which was read with flow cytometry. The results indicated that several compounds—ATXs 30, 33, 35, 37, 38, 41–45, 48–51, and 55—increased the amount of accumulated RHO123, proving their inhibitory effect on P-gp. Also, the ability of the tested compounds (1 or 10 mM, for 48 h) to decrease doxorubicin’s cell-growth-inhibitory effects in K562 and K562Dox cells was evaluated by the sulforhodamine-B assay. As expected, the inhibitory effects obtained in the K562Dox cell line were higher than in the K562 cell line. The most potent cell growth and P-gp inhibitor was the ATX 37 (1-{[2-(diethylamino)ethyl]amino}-4-propoxy-9H-thioxanthen-9-one) [144]. On the other hand, several thioxanthonic derivatives demonstrated P-gp-inhibitory effects, enhancing the therapeutic effects of antibiotics against resistant bacterial strains [79].

Despite all of the current knowledge about this area, DDIs can still happen accidentally in the household, causing harmful outcomes for any person. At the beginning of the century, Barone et al. reported a case of interaction between St. Johns’ wort and cyclosporine in a 29-year-old white woman who had received a multiple-organ transplant. After self-medicating with the plant extract for mood elevation, the prescribed cyclosporine concentrations (100 mg, twice daily) became subtherapeutic (dropping to 155 ng/mL in four weeks), leading to an organ rejection episode. After stopping the St. John’s wort supplementation, the cyclosporine doses reached therapeutic levels again [156].

Table 3 shows some examples of DDIs involving P-gp modulation at the intestinal level, as well as the positive/negative consequences, i.e., reduction in toxicity, lack of therapeutic efficacy, or even toxicity scenarios. Overall, it has been extensively demonstrated that it is possible to reduce the cytotoxicity induced by toxic P-gp substrates in the enterocytes by exposing them simultaneously to P-gp inducers and/or activators.

### 4.2. P-gp Modulation at the Kidney Level

The kidneys’ main function is to carry out the elimination of several compounds, including metabolism products or xenobiotics that are not yet transformed [39,147,168]. In this process, two major steps are involved: glomerular filtration, and tubular secretion. Therefore, the substances that are reabsorbed after the glomerular filtration or the ones that have still not been expelled suffer the tubular secretion phenomenon. Tubular secretion is mostly performed in the kidneys’ proximal tubules, with the help of transporters present in the basolateral and apical membranes of the epithelial proximal tubular cells, in two steps: (a) uptake of compounds from the peritubular fluid into the tubular cells through the basolateral membrane, and (b) efflux of compounds from the tubular cells into the luminal fluid through the apical membrane [39,147,169,170,171].

Among the efflux transporters, P-gp plays a crucial role in the detoxification of several endobiotics and xenobiotics [147]. It is worth emphasizing that, following the confirmation of P-gp’s role in the renal excretion of drugs using established inhibitors, there is a need to investigate other modulators that may influence the elimination of particular substrates. Among the enormous variety of P-gp substrates previously described (Table 2), some are particularly nephrotoxic, including levofloxacin, an antibiotic [172]; tenofovir, an antiviral [39]; cisplatin, an anticancer agent [24]; cyclosporine A, an immunosuppressant [173]; paraquat, a herbicide [91]; and even arsenic, a metal [174]. In the specific case of cisplatin, nephrotoxicity is currently one of the biggest challenges of this drug in therapeutics. Therefore, the inhibition of uptake transporters at the kidney level might be an interesting approach to reduce cisplatin-induced nephrotoxicity [14]. Another possibility would be to increase the expression and/or activity of efflux transporters like P-gp (as cisplatin is a P-gp substrate), thus increasing its renal elimination [137].

Consequently, the use of P-gp inducers/activators, as expected, has been suggested to improve the activity and/or expression of this efflux pump, decreasing the nephrotoxicity induced by these xenobiotics. As previously mentioned, to the best of our knowledge, studies on the induction or activation o P-gp at the kidney level are scarce (Table 4). At the beginning of this century, the group of Tramonti et al. started a line of research on the transport phenomenon performed by P-gp in PTECs. To accomplish this, they started by proving the usefulness of the HK-2 cell line in studying P-gp’s expression, as determined by immunoblotting analysis using the monoclonal antibody C219, and activity, assessed by measuring the transport of the fluorescent probe RHO123. The main results of the study demonstrated that HK-2 cells expressed functional P-gp, the activity and expression of which could be inhibited by known inhibitors, such as verapamil and cyclosporine A [175]. Despite the obtained results, the potential of distinct compounds for the induction or activation of P-gp was not determined in this work. However, Romiti et al. moved towards a more specific characterization of P-gp’s properties in HK-2 cells in the presence of several exogenous or endogenous modulators (e.g., cyclosporine A, vitamin D_3_ [1,25(OH)_2_D_3_], platelet-activating factor, dexamethasone, aldosterone, verapamil, cimetidine, trimethoprim), using RT-PCR and Western blotting to assess the P-gp gene and protein expression, respectively, and RHO123 accumulation assays to assess the P-gp transport activity. Regarding the alterations in P-gp expression, the results showed that cyclosporine A (1 µM), vitamin D_3_ (35 nM), platelet-activating factor (20 µM), dexamethasone (1 µM), and aldosterone (1 µM) significantly induced P-gp expression in 4 days, while the rest of the compounds did not show significant alterations. In terms of P-gp function, incubation with verapamil (10 µM), cyclosporine A (1 µM), vitamin D_3_ (35 nM), and dexamethasone (1 µM) for 2 h significantly decreased the P-gp function, while no alterations were observed for the remaining compounds. In this study, activators were not detected and, additionally, there was no assessment of the extent to which the observed increases in expression were reflected in greater activity. The impact of the observed P-gp modulation on the cytotoxicity of P-gp substrates was also evaluated. The researchers evaluated the cell viability in the absence/presence of dexamethasone (1 µM), cyclosporine A (1 µM), and vitamin D_3_ (35 nM) for 4 days, with subsequent exposure to cyclosporine A (up to 100 µM, for 36 h), by trypan blue dye exclusion tests and MTT assays. They showed that HK-2 cells pretreated with dexamethasone, cyclosporine A, and vitamin D3 were protected against cyclosporine A-induced cytotoxicity (itself), and this effect was reversed by verapamil (a known P-gp inhibitor) [176].

Many plant-derived compounds can also affect P-gp activity, leading to potential herb–drug interactions. Using a similar methodology—RT-PCR and Western blotting to assess P-gp gene and protein expression, respectively, and calcein-AM assay to evaluate P-gp transport activity—the effects of grapefruit juice and two related flavonoids (kaempferol and naringenin) on P-gp expression/activity were investigated. Grapefruit juice, kaempferol, and naringenin (5%, for 3 days) produced inhibitory effects on P-gp expression/activity in HK-2 cells, increasing the cytotoxicity induced by the known P-gp substrates cyclosporine A and vinblastine [177]. An inhibitory effect on P-gp transport activity has also been shown for n-hexane root extracts from *Echinacea pallida*, *Echinacea angustifolia*, and *Echinacea purpurea*, since they decreased the efflux of its substrate (calcein-AM) from HK-2 cells into the cell culture medium by twofold, and in a concentration-dependent manner [178]. On the other hand, the group also investigated the effects on P-gp expression and function caused by commercially obtained preparations of devil’s claw (a plant from Southern Africa used in traditional medicine and, more recently, as an anti-inflammatory and pain-relieving natural medicine) and harpagoside, an iridoid glycoside present in the secondary roots of the plant. Researchers used the calcein-AM test and Western blotting techniques to assess P-gp activity and expression, respectively. The results showed that extracts of devil’s claw (262.4–303.6 µg/mL, for 1 h) caused an increase in intracellular calcein fluorescence as compared with the control, proving their inhibitory effect on P-gp activity, while harpagoside (0.5 to 200 µM, for 1 h) did not cause any effect. In terms of the P-gp expression, the preparations of devil’s claw (4 or 400 µg/mL, for 72 h) and harpagoside (0–200 µM, for 72 h) showed a dose-dependent upregulation of P-gp. However, no correlation was found between the harpagoside content in the extracts and the relative increase in P-gp expression, suggesting that this phenomenon could occur due to other unknown compounds of the preparations [179].

Other studies demonstrated changes in P-gp expression/activity in both Caco-2 and HK-2 cell lines, caused by exposure to mango (*Mangifera indica* L.) stem bark extracts and derived phenols, using Western blot analysis and the RHO123 and calcein-AM assays, highlighting the potential existence of herb–drug interactions. The results indicated that the mango stem bark extract (10–200 µg/mL, for 72 h) caused a concentration-dependent decrease in P-gp expression, while cells exposed to mangiferin (10–200 µM, for 72 h) showed a significant and concentration- and time-dependent increase in P-gp expression levels. In terms of the evaluation of P-gp activity, the results showed an increase in calcein-AM accumulation when the cells were exposed to mango stem bark extract (50 and 200 µg/mL, for 72 h), while the exposure to mangiferin (10–200 µM, for 72 h) caused a decrease in the accumulation of calcein-AM. In this way, the authors concluded that the mango stem bark extract had inhibitory effects on P-gp, while mangiferin had the reverse effect, possibly being a P-gp inducer. With the exposure to norathyriol (5 µM, for 72 h), induction of P-gp was observed, and this effect decreased at higher concentrations [180]. *Zuccagnia punctata* extracts (3,7-dihydroxyflavone and 20,40-dihydroxychalcone) (5 mg/mL, for 3 days) also inhibited P-gp, since increased RHO123 contents were detected in HK-2 cells after exposure, showing their potential role in the modulation of MDR [181].

Li et al. successfully found a way to decrease the nephrotoxicity caused by triptolide, a traditional herbal medicine isolated from *Tripterygium Wilford Hook. f.*, which frequently exhibits adverse reactions. For this, HK-2 cells were exposed to glycyrrhetinic acid (0–100 µM, for 8, 12 and 24 h), a potential P-gp modulator; triptolide (0–5000 nM, for 6, 12 and 24 h), a toxic P-gp substrate; and verapamil (20 µM, for 2 h) or MK571 (50 µM, for 2 h), known inhibitors of P-gp and MRP2, respectively (in this way, it is possible to distinguish which transporter is truly exporting the toxic compound). With the help of ultra-performance liquid chromatography–electrospray ionization–mass spectrometry (UPLC-ESI-MS), it was observed that the accumulation of triptolide was significantly decreased in the presence of glycyrrhetinic acid, thus demonstrating the potential of glycyrrhetinic acid in detoxifying HK-2 cells against triptolide. Additionally, exposure to verapamil or MK571 caused a significant increase in the intracellular accumulation of triptolide. Later, the authors showed that the P-gp and MRP2 expression levels were elevated in the presence of glycyrrhetinic acid. Therefore, they concluded that glycyrrhetinic acid is capable of protecting HK-2 cells against triptolide-induced cytotoxicity—an effect that seems to be mediated by both P-gp and MRP2 [182].

After these studies investigating the *in vitro* expression/activity of P-gp at the kidney level, several researchers carried out some *ex vivo*/*in vivo* studies aiming to discover other compounds that can act as P-gp modulators. Chow et al. investigated whether the vitamin D receptor could be involved in increased P-gp expression in the liver and kidneys of rats, after treatment with dihydroxyvitamin D_3_ (1,25(OH)_2_D_3_] (2.5 µg/kg every other day, for 8 days, i.p.)), the natural ligand of the vitamin D receptor. Renal and cerebral P-gp protein levels were significantly increased (3.45-fold), causing an increase in renal (74%) and total-body (34%) clearance of digoxin [183]. In another study, the possible interaction between cilastatin and P-gp, along with its effects on vancomycin-induced nephrotoxicity, was evaluated using the HK-2 cell line for *in vitro* studies and male C57BL/6J mice to perform *in vivo* studies. When exposed only to vancomycin (HK-2 cells: 2 or 4 mM, for 72 h; C57BL/6 mice: 400 or 600 mg/kg/day, for 7 days), HK-2 cells demonstrated a reduced expression and function of P-gp, while C57BL/6 mice presented an increase in the serum blood urea nitrogen and creatinine levels, indicating decreased renal function. In the presence of cilastatin (200 µg/mL, for 0–72 h), the *in vitro* results showed a decreased vancomycin-induced P-gp suppression, among other effects, including decreased reactive oxygen species (ROS) production and apoptosis, proving the protective role of cilastatin against vancomycin-induced nephrotoxicity. *In vivo*, the administration of cilastatin (300 mg/kg/day, for 7 days) significantly decreased the vancomycin concentrations in the blood and kidneys, suggesting increased elimination of vancomycin. Therefore, the authors concluded that the suppression of vancomycin-induced nephrotoxicity was at least partially due to the important role of P-gp, since cilastatin prevented the decrease in P-gp levels caused by vancomycin, which resulted in less accumulation of vancomycin in the blood and kidneys [184].

In order to decrease the cisplatin-induced nephrotoxicity, the P-gp modulation mediated by omeprazole, a proton pump inhibitor, was also studied *in vitro* (HK-2 cells) and *in vivo* (male Sprague Dawley rat kidneys). HK-2 cells were exposed to cisplatin (0.02–200 nM) in the presence/absence of omeprazole (0.4–4000 µg/mL) for 12 h, 24 h, and 48 h, and then the cell viability was measured by the MTT reduction assay. On the other hand, rats were exposed to cisplatin (15 mg/kg, one dose, i.p.) alone, or with co-administration of omeprazole (1.8 or 3.6 mg/kg/day, for 5 days, i.p.), and sampled for the determination of plasma blood urea nitrogen and creatinine levels. The authors found that, *in vitro*, P-gp levels were significantly elevated in HK-2 cells exposed to both drugs when compared with the exposure to cisplatin alone, resulting in a significant reduction in cisplatin-induced cytotoxicity upon simultaneous exposure to both drugs. The *in vivo* results indicated lower levels of plasma blood urea nitrogen and creatinine, as well as a reduction in the necrosis of epithelial cells, cellular vacuolization, and cast formation in the case of co-administration of cisplatin and omeprazole, when compared to the administration of cisplatin alone, suggesting that omeprazole actually reduced the cisplatin-induced nephrotoxicity. On the other hand, the levels of OCT2, an uptake transporter, were decreased in the presence of omeprazole, which caused a lower entry of cisplatin into PTECs, also helpful in explaining the obtained results. Therefore, the acquired data helped to conclude that omeprazole prevented cisplatin-induced nephrotoxicity via mechanisms potentially involving both P-gp and OCT2 [137].

Kanado et al. evaluated the ability of 17b-estradiol to modulate P-gp expression and activity *in vitro* and *in vivo*. The *in vitro* findings showed that P-gp expression was induced by 17b-estradiol (10 nM, for 24 h) in cultured human PTECs, consequently resulting in increased export of digoxin, a classic P-gp substrate. *In vivo*, female and male ICR mice were injected with digoxin (0.625 mg/kg, i.v.), and it was observed by LC-MS-MS that the renal clearance of digoxin in female mice was approximately twofold higher than that observed in male mice. Using RT-PCR, the expression of murine P-gp was then evaluated, with the P-gp mRNA levels also being higher in female than in male mice, suggesting that 17b-estradiol has an induction effect on P-gp expression, since it is present in higher quantities in females [185]. *In vivo*, Semeniuk et al. evaluated the effects of the phytoestrogen genistein on P-gp expression in the liver, kidneys, and ileum of rats. However, they observed that genistein (5 mg/kg/day for 3 consecutive days, subcutaneously, 24 h before the analysis) significantly increased hepatic P-gp expression and activity, but the renal and intestinal P-gp levels remained unchanged [186].

Table 4 summarizes the studies performed in both *in vitro* and *in vivo* kidney models in which the positive or negative modulation of P-gp has been demonstrated, resulting in changes in the therapeutic effect and/or toxicity of xenobiotics. Overall, as evident from the previously mentioned studies, there are considerably more studies identifying P-gp inhibitors at the kidney level compared to those involving P-gp inducers and/or activators. Furthermore, while a limited number of P-gp inducers and activators have been identified at the kidney level, there is a scarcity of studies dedicated to exploring their potential in reducing drug-induced nephrotoxicity. Nevertheless, and as already demonstrated for the intestines, in the presence of P-gp inducers/activators, the P-gp present in renal PTECs can prevent drug-induced nephrotoxicity by enhancing the efficient export of toxic substrates. This mechanistic approach thus deserves to be further explored and could potentially be employed as a therapeutic strategy to mitigate drug-induced kidney failure.

**Table 4:** Examples of P-glycoprotein-mediated drug–drug interactions occurring in renal tubular cells.

P-gp Substrate	Experimental Conditions	Modulation Type	Alterations in Substrate Therapeutic Effect/Toxicity	Experimental Models	References
**Cisplatin**	*In vitro:* 0.02–200 nM, for 24 h; *in vivo:* 15 mg/kg, i.p.	**P-gp induction** by omeprazole (along with OCT2 inhibition) *In vitro:* 0.4–4000 µg/mL, for 24 h *In vivo:* 1.8 or 3.6 mg/kg/day, i.p., for 5 days	Decreased cisplatin-induced nephrotoxicity Enhanced cell viability; diminished intracellular ROS generation and membrane damage	*In vitro*, in HK-2 cells Protein expression: Western blot, immunofluorescence, and immunohistochemistry assay *In vivo*, in male Sprague Dawley rat kidneys	[137]
**Cyclosporine A**	1–20 µM, for 36 h	**P-gp induction** by: Dexamethasone (1 µM, for 4 days) Cyclosporine A (1 µM, for 4 days) 1,25(OH)2D3 (35 nM, for 4 days)	Lower cyclosporine A-induced cytotoxicity	*In vitro*, in HK-2 cells Cell viability: trypan blue dye exclusion test and MTT assay	[176]
	0–80 µM, for 36 h	**P-gp inhibition** by grapefruit juice (5%, for 3 days)	Higher cyclosporine A-induced cytotoxicity	*In vitro*, in HK-2 cells Cell viability: trypan blue dye exclusion test and WST-1 colorimetric test	[177]
	0–80 µM, for 24 h	**P-gp inhibition** by *Zuccagnia punctata* extract or 3,7-dihydroxyflavone (5 mg/mL, for 3 days)	Higher cyclosporine A-induced cytotoxicity	*In vitro*, in HK-2 cells Cell viability: NR assay and trypan blue exclusion test	[181]
**Digoxin**	0.75 mg, for 3 days, orally	**P-gp inhibition** by clarithromycin (2 × 250 mg/day, for 3 days, orally)	Higher rate of adverse effects Reduced non-glomerular renal clearance of digoxin	Randomized, placebo-controlled, double-blind crossover design applied to 12 healthy men Concentrations of digoxin in plasma and urine: radioimmunoassay	[135]
	0.1 mg/kg, i.v.	**P-gp induction** by 1,25(OH)2D3 (2.5 µg/kg every other day for 8 days, i.p.)	Significant increases in renal and cerebral P-gp levels (3.45-fold), and in the renal (74%) and total-body (34%) clearance of digoxin	*In vivo*, in fxr(−/−) mice	[183]
	0.625 mg/kg, i.v.	**P-gp induction** by 17b-estradiol *In vitro*: 10 nM, for 24 h	*In vitro*: 17b-estradiol induced the expression of P-gp in cultured human PTECs *In vivo*: higher renal clearance of digoxin and mRNA expression in female mice (higher levels of 17b-estradiol)	*In vitro*, in renal PTECs P-gp expression: RT-PCR *In vivo*, in female and male ICR mice Quantification of digoxin by LC-MS-MS	[185]
**Vancomycin**	*In vivo*: 2 or 4 mM for 72 h; *in vivo:* 400 or 600 mg/kg/day, for 7 days	**P-gp induction** by cilastatin *In vitro:* 200 µg/mL, for 0–72 h *In vivo:* 300 mg/kg/day, for 7 days	*In vitro:* Lower vancomycin-induced cytotoxicity *In vivo:* Significant decrease in vancomycin levels in the blood and kidneys	*In vitro*, in HK-2 cells Cell viability: MTT assay *In vivo*, in male C57BL/6J mice Quantification of vancomycin: fluorescence polarization immunoassay	[184]
**Triptolide**	0–5000 nM, for 6, 12 and 24 h	**P-gp induction** by glycyrrhetinic acid (0–100 µM, for 8, 12 and 24 h)	Lower glycyrrhetinic-acid-induced cytotoxicity	*In vitro*, in HK-2 cells Cell viability: MTT assay Quantification of triptolide by UPLC-ESI-MS	[182]

Legend: HK-2: human kidney-2; i.p.: intraperitoneal; i.v.: intravenous; LC-MS-MS: liquid chromatography–tandem mass spectrometry; MTT: 3-(4,5-dimethylthiazol-2-yl)-2,5-diphenyltetrazoliumbromide; NR: neutral red; P-gp: P-glycoprotein; PTECs: proximal tubular epithelial cells; ROS: reactive oxygen species; RT-PCR: reverse-transcription polymerase chain reaction; UPLC-ESI-MS: ultra-performance liquid chromatography–electrospray ionization–tandem mass spectrometry.

## 5. Concluding Remarks

The scientific community has made great progress in developing new techniques and/or optimizing classic assays to assess P-gp activity/expression and drug-mediated P-gp modulation. Currently, a great variety of techniques are available in this field, making it possible to choose the most adequate strategy for achieving the goals of our research. Each type of research model presents several advantages but also certain limitations that need to be addressed. For instance, computational and *in vitro* studies offer a high throughput of results in identifying P-gp substrates and modulators, although interpreting the results and extrapolating the data to the human body can be challenging. On the other hand, *ex vivo* and *in vivo* studies provide a better understanding of xenobiotics’ pharmacokinetics and toxicokinetics, but the screening of P-gp substrates and modulators can be delicate due to ethical reasons. Nevertheless, regardless of the selected method(s), it is crucial to meticulously interpret the obtained results, considering several factors that can potentially impact P-gp transport activity. Additionally, it is important to be aware of the presence of compounds that can simultaneously function as both P-gp substrates and modulators, as well as those that can also act as CYP450 substrates.

P-gp is a non-specific efflux transporter located in important barrier/excretory tissues, with a vast variety of substrates. Between them, some can induce cytotoxicity, which makes P-gp a key player in the detoxification of xenobiotics. Worldwide, the possibility of using DDIs as a potential therapeutic strategy to overcome drug-induced nephrotoxicity and/or to increase drug elimination at the intestinal level has been widely investigated. Indeed, P-gp inducers and/or activators will potentially increase P-gp expression and/or activity, respectively, causing an increase in P-gp-mediated transport of its substrates from the PTECs into the urine or from the enterocytes into the feces. Consequently, the accumulation of toxic substrates within kidney cells will decrease, thus reducing their nephrotoxic effects, while at the intestinal level a decrease in the absorption of xenobiotics through the enterocytes will occur, reducing the xenobiotics’ bioavailability and systemic toxicity. Nonetheless, it remains crucial to collaborate and invest efforts in the study of P-gp modulation at the kidney level. This field of research is still emerging, with relatively few studies having been conducted. However, it represents a viable and promising approach to mitigate the cytotoxicity of nephrotoxic agents by either inducing or activating this remarkably important efflux pump.

## Figures and Tables

**Table 1:** Advantages and limitations of the different types of research models used in the assessment of P-gp modulation.

Research Models	Advantages	Limitations	References
*In silico*	Allows for the simulation of biological systems; represents a reliable alternative to excessive animal use and other experimental techniques; enables larger and faster screenings to identify P-gp modulators/substrates; is less expensive, laborious, and time-consuming	Complexity of the methods; method selection can be difficult; difficult extrapolation to the *in vitro*, *ex vivo*, and *in vivo* experimental approaches; need for the acquisition of specific and sometimes expensive software	[33,49]
*In vitro*	Possibility of screening for a large number of substrates and/or modulators; possibility of testing several experimental conditions at the same time; allows for the assessment of the IC_50_ (for inhibitors), kinetic studies, etc.; allows for a high-throughput mode	Difficult clarification of the precise targets of xenobiotics, given the expression of multiple transporters in a particular cell line; difficult extrapolation of the results to the human body; laborious, time-consuming, and expensive; the cells need to be maintained under aseptic cell culture conditions prior to use	[38,48]
*Ex vivo*	Higher accuracy in the determination of the transporter’s function in absorption and/or elimination processes; allows for a simple understanding of the transporter’s role in one specific organ; allows for the evaluation of the interactions between transporters and metabolic enzymes	Difficult clarification of the modulation of one specific efflux transporter; requirement of ethics committees’ approval; requirement of surgical skills and equipment; expensive, laborious, and time-consuming; difficult data extrapolation to humans	[38]
*In vivo*	Better perspective on the (pharmaco)toxicokinetic characteristics; easier extrapolation to the human organism	Requirement of ethics committees’ approval; does not permit screenings of a large number of compounds and/or distinct experimental conditions	[48]

**Table 2:** Examples of P-glycoprotein substrates, inhibitors, inducers, and activators [3,18,24,38,114,124,133,144,145,146,147,148].

**Substrates** (actively transported by P-gp)	**Analgesics**: morphine; **anti-arrhythmics**: amiodarone, propafenone, quinidine; **anti-alcoholism drugs**: disulfiram; **anticancer drugs**: bisantrene, catharanthine, cisplatin, daunorubicin, docetaxel, doxorubicin, etoposide, irinotecan, mitoxantrone, paclitaxel, teniposide, topotecan, vinblastine, vincristine; **anticonvulsants**: phenobarbital, phenytoin; **antidepressants**: amitriptyline, nortriptyline, doxepin, venlafaxine, paroxetine; **antidiarrheal agents**: loperamide; **anti-inflammatory agents**: flunisolide; **antiemetics**: ondansetron, domperidone; **antiepileptics**: phenytoin, felbamate, topiramate, carbamazepine, lamotrigine, phenobarbital, gabapentin, topiramate; **anti-gout agents**: colchicine; **antihistamines**: terfenadine, fexofenadine; **anti-hypertensives**: debrisoquine, reserpine, propranolol, celiprolol, diltiazem, losartan, talinolol, prazosin; **antimicrobial agents**: actinomycin D, amoxicillin, clarithromycin, doxycycline, erythromycin, fluoroquinolones, gramicidin, grepafloxacin A, itraconazole, ketoconazole, levofloxacin, rifampin, sparfloxacin, valinomycin, tetracyclines, tetracycline; **anti-tuberculous agents**: erythromycin, rifampin; **anthelmintics**: abamectin, ivermectin; **calcium channel blockers**: verapamil, nifedipine, azidopine, diltiazem, nicardipin; **cardiac glycosides**: digitoxin, digoxin, quinidine; **calmodulin antagonists**: trifluoperazine, chlorpromazine, trans-flupentixol; **hepatitis C treatments**: ledipasvir/sofosbuvir; histamine H2 receptor antagonists: cimetidine; **HIV protease inhibitors**: ritonavir, saquinavir, nelfinavir, amprenavir, indinavir, maraviroc, darunavir; **HMG-CoA reductase inhibitors**: lovastatin, simvastatin; **immunosuppressive agents**: sirolimus, valspodar, cyclosporine A, tacrolimus (FK506); **fluorescent compounds**: berberine, calcein-AM, Hoechst 33342, rhodamine 123; **natural products**: flavonoids, curcuminoids, rhizome extract; **opioids**: loperamide, morphine, pentazocine; **pesticides**: methyl parathion, endosulfan, paraquat, cypermethrin, fenvalerate; **steroids**: budesonide, dexamethasone, hydrocortisone, corticosterone, cortisol, aldosterone, methylprednisolone, triamcinolone acetonide; **tyrosine kinase inhibitors**: imatinib mesylate, gefitinib, nilotinib, tandutinib; **thrombin inhibitors**: dabigatran
**Inhibitors** (block P-gp-mediated transport by different mechanisms)	**First-generation**: verapamil, cyclosporine A, nifedipine, quinidine, quinine, amiodarone, tamoxifen detergents; **second-generation**: R-verapamil, PSC833, dexniguldipine, valspodar, elacridar, biricodar, dexverapamil, dofequine fumarate; **third-generation**: ontogen (OC 144-093), zosuquidar, tariquidar, elacridar, laniquidar, biricodar; **fourth-generation**: anti-arrhythmics: propafenone; hepatitis C treatments: dasabuvir, ledipasvir, paritaprevir, ritonavir; natural products: flavonoids like tangeretin, sinensetin, baicalein, quercetin, ellipticine, cnidiadin, praeruptorin, capsaicin; surfactants: sodium dodecyl sulfate, Tween-20 and Span-80, and lipids; others: clarithromycin, itraconazole
**Inducers** (increase P-gp protein expression by prompting the transcription of the *MDR1/ABCB1* gene)	Abacavir, actinomycin D, aflatoxin B1, aldosterone, ambrisentan, amiodarone, amprenavir, m-amsacrine, apigenin, artemisinin, asiatic acid, atazanavir, atorvastatin, avermectin, beclomethasone, benzo(a)pyrene, benzo(e)pyrene, berberine, betamethasone, bilirubin, bosentan, bromocriptine, budesonide, caffeine, cadmium chloride, capsaicin, carbamazepine, catechin, celiprolol, cembratriene, R-cetirizine, CITCO, chlorambucil, cholate, chrysin, ciclesonide, cisplatin, clotrimazole, colchicine, corticosterone, *curcuma* family extracts, curcumin, cyanidin, cyclophosphamide, cyclosporine A, cytarabine, daidzein daunorubicin, daurunavir, depsipeptide (FR901228, FK228, NSC630176), desvenlafaxine, dexamethasone, diclofenac, digoxin, dihydroxylated xanthones, 1α,25-dihydroxyvitamin D_3_, diltiazem, dimethylformamide, 6,16α-dimethylpregnenolone, dimethyl sulfoxide, docetaxel, doxorubicin, doxycycline, efavirenz, emetined, epigallocatechin-3-gallate, epirubicin, eriodictyol, erythromycin, β-estradiol, ethinylestradiol, etoposide, fenbufen, flavone, 5-fluorouracil, flutamide, fluticasone, genistein, ginkgolides a and b, hydroxyurea, hyperforin, hypericin, *Hypericum perforatum* extracts (Saint John’s wort), idarubicin, ifosfamide, indinavir, indomethacin, insulin, isosafrole, isoxanthohumol, ivermectin, lopinavir, LY191401, mangiferin, meloxicam, mepirizole, methotrexate, methylprednisolone, midazolam, mifepristone, mitoxantrone, morphine, mx2, myricetin, naringenin, nefazodone, nelfinavir, nevirapine, nicardipine, nifedipine, nimesulide, norathyriol, oleocanthal, ouabain, oxycodone, paclitaxel, parthenolide, pentylenetetrazole, phenobarbital, phenothiazine, phenytoin, phorbol 12-myristate 13-acetate, piperine, platelet-activating factor, prednisolone, 5β-pregnane-3,20-dione, pregnenolone-16α-carbonitrile, probenecid propranolol, quercetin, quinidine, rapamycin or sirolimus, rescinnamine, reserpine, retinoic acid,, rhinacanthin-C, rifabutin, rifampicin and derivatives, rilpivirinem, ritonavir, saquinavir, small-molecule tyrosine kinase inhibitors (e.g., erlotinib, gefitinib, nilotinib, sorafenib, vandetanib), sildenafil, sodium arsenite, sodium butyrate, spironolactone, SR12813, sulindac, tacrolimus, tadalafil, tamoxifen, tangeretin, talazoparib, taurocholate, taxifolin, TCDD, thioxanthonic derivatives (e.g., 1-(propan-2-ylamino)-4-propoxy-9*H*-thioxanthen-9-one (TX5)), γ-tocotrienol, topotecan, trazodone, triactyloleandomycin, trichostatin A, trimethoxybenzoylyohimbine, venlafaxine, verapamil, vinblastine, vincristine [3], tetrahydrocurcumin
**Activators** (promote an immediate increase in P-gp transport activity without interfering with P-gp protein expression)	Dihydroxylated xanthones (X1–X5): 3,4-dihydroxy-9*H*-xanthen-9-one, 1,2-dihydroxy-9*H*-xanthen-9-one, 1,3-dihydroxy-9*H*-xanthen-9-one, 2,3-dihydroxy-9*H*-xanthen-9-one, 3,6-dihydroxy-9*H*-xanthen-9-one; thioxanthonic derivatives (TX1-TX5): 1-[(3-hydroxypropyl)amino]-4-propoxy-9*H*-thioxanthen-9-one, 1-chloro-4-hydroxy-9*H*-thioxanthen-9-one, 1-{[2-(1,3-benzodioxol-5-yl) ethyl]amino}-4-propoxy-9*H*-thioxanthen-9-one, 1-[(2-methylpropyl) amino]-4-propoxy-9*H*-thioxanthen-9-one, 1-(propan-2-ylamino)-4-propoxy-9*H*-thioxanthen-9-one; aminated thioxanthones (ATX1-ATX8): (S)-1-((1-hydroxypropan-2-yl)amino)-4-propoxy-9*H*-thioxanthen-9-one, (R)-1-((1-hydroxypropan-2-yl)amino)-4-propoxy-9*H*-thioxanthen-9-one, (S)-1-((2-hydroxypropyl)amino)-4-propoxy-9*H*-thioxanthen-9-one, (R)-1-((2-hydroxypropyl)amino)-4-propoxy-9*H*-thioxanthen-9-one, (S)-1-((1-hydroxy-4-methylpentan-2-yl)amino)-4-propoxy-9*H*-thioxanthen-9-one, (R)-1-((1-hydroxy-4-methylpentan-2-yl)amino)-4-propoxy-9*H*-thioxanthen-9-one, (S)-1-((1-hydroxy-3-methylbutan-2-yl)amino)-4-propoxy-9*H*-thioxanthen-9-one, (R)-1-((1-hydroxy-3-methylbutan-2-yl)amino)-4-propoxy-9*H*-thioxanthen-9-one; oxygenated xanthones (OX1-OX2, OX4-OX6): 3,4-dimethoxy-1-methyl-9*H*-xanthen-9-one, 1-(dibromomethyl)-3,4-dimethoxy-9*H*-xanthen-9-one, 4-hydroxy-3-methoxy-9-oxo-9*H*-xanthene-1-carbaldehyde, 3,4-dimethoxy-9-oxo-9*H*-xanthene-1-carbaldehyde, 1-(hydroxymethyl)-3,4-dimethoxy-9*H*-xanthen-9-one

Legend: ATXs: aminated thioxanthones; HMG-CoA reductase: 3-hydroxy-3-methyl-glutaryl-coenzyme A reductase; OXs: oxygenated xanthones; TCDD: 2,3,7,8-tetraclorodibenzo-p-dioxina; TXs: thioxanthonic derivatives; Xs: dihydroxylated xanthones.

**Table 3:** Examples of P-glycoprotein-mediated drug–drug interactions occurring in enterocytes.

P-gp Substrate	Experimental Conditions	Modulation Type	Alterations in Substrate Therapeutic Effect/Toxicity	Experimental Models	References
**[3H]-carbamazepine** **[3H]-dexamethasone** **[3H]-imipramine** **[3H]-lansoprazole** **[3H]-prazosin** **[3H]-quinidine** **[3H]-rifampicin** **[3H]-ritonavir** **[3H]-tamoxifen**	1 mCi, for 30 min	**P-gp induction** by tetrahydroisoquinoline derivative 8b (N-{4′-[(6,7-dimethoxy-1,2,3,4-tetrahydroisoquinolin-2-yl) methyl]-[1,1′-biphenyl]-4-yl}-4-methoxy-N-(4-methoxy- benzenesulfonyl)benzene-1-sulfonamide, 100 nM, for 2 h)	Higher efflux transport of P-gp substrates	*In vitro*, in Caco-2 cells Efflux/accumulation assay Substrates’ radioactivity measured by liquid scintillation	[157]
**Colchicine**	0.1 mM, for 0–120 min	**P-gp inhibition**: *In vitro*, grapefruit juice (1–10%), 6′,7′-dihydroxybergamottin (1–500 µM), naringin (100–2000 µM), naringenin (10–500 µM), verapamil and quinidine (0.01, 0.05 and 0.1 mM), for 30 min; *In situ*, grapefruit juice (10%, for 1 h)	Inhibition of efflux transport of colchicine, proven by the increase in AP–BL permeability and decrease in BL–AP Higher oral bioavailability of colchicine	*In vitro*, in Caco-2 cell monolayers AP–BL and BL–AP directions in the absence/presence of known P-gp inhibitors (verapamil and quinidine) *In situ*, in male albino Wistar rats Single-pass intestinal perfusion studies, in both the jejunum and ileum	[158]
**Clopidogrel**	10 µM, for 20–120 min	**P-gp inhibition** by omeprazole (6–60 µM), cyclosporin (2–20 µM), verapamil (25–250 µM), quinidine (20–200 µM), and elacridar (0.12–1.2 µM), for 20–120 min	Higher intracellular accumulation of clopidogrel Higher bioavailability of clopidogrel	*In vitro*, in Caco-2 cells Quantification of clopidogrel in LC-MS-MS	[150]
**Cyclosporine A**	100 mg, twice daily	**P-gp induction** by St. Johns’ wort (300–600 mg/day)	Lower absorption of cyclosporine A Lower serum levels of cyclosporine	Case report, in a 29-year-old white woman Quantification of serum cyclosporine A levels	[156]
**Digoxin**	0.02 mg/kg, orally	**P-gp inhibition** by quercetin (40 or 50 mg/kg, orally)	Higher C_max_ and AUC of digoxin	*In vivo*, in male Yorkshire pigs Quantification of serum digoxin levels through fluorescence polarization immunoassays	[159]
	0.04 mg/mL, orally	**P-gp inhibition** by montmorillonite–surfactant hybrid particles (5.0 mL/kg, orally)	Higher C_max_ and AUC of digoxin	*In vivo*, in male Sprague Dawley rats LC-MS-MS: blood levels of digoxin	[160]
	0.1 µM of [3H]-digoxin, for 30 min	**P-gp inhibition**: Berberine formulation with P85 (0.1% and 1%, for 30 min) and Tween 80 (0.1% and 1%, for 30 min)	Lower P-gp-mediated efflux of digoxin Higher intestinal accumulation of digoxin	*In vitro*, in LLC-PK1-P-gp cells [3H]-digoxin radioactivity measured by a liquid scintillation counter	[161]
	0.25 mg/kg, orally	**P-gp activation** by thioxanthone 5 (30 mg/kg, orally)	Lower AUC of digoxin	*In vivo*, in adult male Wistar Han rats Digoxin plasma levels measured on an AutoAnalyzer	[122]
	0.2 mg/kg, orally	**P-gp inhibition** by verapamil (25 mg/kg, orally)	Higher C_max_ and AUC of digoxin	*In vivo*, in male Sprague Dawley rats LC-MS-MS: blood levels of digoxin	[162]
**Doxorubicin**	1.0 µM, every two weeks in the cell culture medium	**P-gp inhibition** by aminated thioxanthones (1 or 10 µM, for 48 h)	Higher cell-growth-inhibitory effect	*In vitro*, in K562 and K562Dox cells Sulforhodamine-B assay: measurement of the cell-growth-inhibitory effect	[144]
**Enrofloxacin**	10 mg/kg, orally	**P-gp induction** by quercetin (15 and 60 mg/kg, for 5 days, orally)	Higher P-gp-mediated function Lower AUC, peak concentration, lower intestinal absorption; higher clearance	*In vivo*, in Arbor Acres chickens Quantification of enrofloxacin plasma levels through HPLC	[163]
**Fexofenadine**	50 µM, for 15 min	**P-gp inhibition** by *Maytenus ilicifolia* (obtained by infusion or turbo-extraction using hydroacetonic solvent, 200 µg/mL, for 30 min)	Lower intestinal metabolism Lower transport of drugs	*In vitro*, in Caco-2 cells Quantification of fexofenadine through HPLC	[164]
**Glibenclamide**	*Ex vivo*: 1 mg/mL, for 2 h; *in vivo*: 3.6 mg/kg, orally, for 8 h	**P-gp inhibition** by carbamazepine (*ex vivo*: 25 mM, for 2 h*; in vivo*: 90 mg/kg, orally, for 1 + 8 h)	*Ex vivo*: lower accumulation of glibenclamide *In vivo*: higher absorption of glibenclamide	*Ex vivo*, in ileal sacs of albino rats *In vivo*, in albino rats Quantification of glibenclamide through HPLC	[131]
**Ivermectin**	*Ex vivo*: 3 µM; *in vivo*: 200 µg/kg, subcutaneously	**P-gp inhibition** by itraconazole (*ex vivo*: 10 µM; *in vivo*: 5 mg, two doses, subcutaneously)	*Ex vivo*: higher levels of ivermectin *In vivo:* higher concentrations of ivermectin in plasma and gastrointestinal tissues; enhanced ivermectin absorption	*Ex vivo*, in everted ileal sacs *In vivo*, in male Wistar rats Quantification of ivermectin levels using HPLC	[130]
	*In vitro*: 10 µM, for 2–24 h; *in vivo*: 0.2 mg/kg, orally	**P-gp inhibition** by oleic acid (*In vitro*: 500 µM, for 2–24 h; *in vivo*: 1 g/kg, orally)	*Ex vivo*: higher ivermectin levels *In vivo*: higher concentrations of ivermectin in plasma and intestinal mucosa	*In vitro*, in Caco-2 cells *In vivo*, in wild-type mice Quantification of ivermectin levels using HPLC	[165]
**Paraquat**	0–5000 µM, for 24 h	**P-gp induction** by doxorubicin (0–100 µM, for 24 h)	Lower paraquat-induced cytotoxicity	*In vitro*, in Caco-2 cells Cellular viability: MTT assays	[90]
	100–5000 µM, for 24 h	**P-gp induction** by doxorubicin (10, 50 and 100 µM, for 18 h)	Lower paraquat-induced cytotoxicity Lower intracellular accumulation of paraquat	*In vitro*, in Caco-2 cells Cellular viability: MTT assays Quantification of intracellular accumulation of paraquat: GC–IT-MS analyses	[94]
	0.5–50 mM, for 48 h	**P-gp induction** by reduced rifampicin (10 µM, for 24, 48 and 72 h)	Lower paraquat-induced cytotoxicity	*In vitro*, in RBE4 cells Cellular viability: NR uptake assay	[114]
	0–7500 µM, for 24 h	**P-gp induction** by dihydroxylated xanthones 1–5 (20 µM, for 24 h)	Lower paraquat-induced cytotoxicity	*In vitro*, in Caco-2 cells Cellular viability: NR uptake assay	[91]
	0–7500 µM, for 24 h	**P-gp induction** by thioxanthones 1–5 (20 µM, for 24 h)	Lower paraquat-induced cytotoxicity	*In vitro*, in Caco-2 cells Cellular viability: NR uptake assay	[83]
	250–2500 µM, for 24 h	**P-gp induction** by oxygenated xanthones 1–6 (20 µM, for 24 h)	Lower paraquat-induced cytotoxicity	*In vitro*, in Caco-2 cells Cellular viability: NR uptake assay	[21]
**Paclitaxel**	40–70 mg/kg, orally	**P-gp inhibition** by flavonoid FM04 (45 mg/kg, orally)	Higher intestinal absorption of paclitaxel; higher AUC	*In vivo*, in male Sprague Dawley rats Quantification of paclitaxel using UPLC-MSMS	[166]
**Mitoxantrone**	0–150 µM, for 24 h	**P-gp induction** by oxygenated xanthones 1–6 (20 µM, for 24 h)	Lower mitoxantrone-induced cytotoxicity	*In vitro*, in SW480 cells and Caco-2 cells Cellular viability: NR uptake assay	[113]
**Talinolol**	20 mg/kg, i.p.	**P-gp inhibition** by zosuquidar (30 mg/kg, i.p.)	Higher AUC_0–5 h_ and AUC_total_	*In vivo*, in C57BL/6 J mice Expression: PCR and Western blot techniques Quantification of talinolol through HPLC-UV	[167]

Legend: AP–BL: apical-to-basal; AUC: area under the curve; BL–AP: basal-to-apical; Cmax: maximum concentration; GC–IT-MS: gas chromatography–ion trap mass spectrometry; HPLC: high-performance liquid chromatography; HPLC-UV: HPLC–ultraviolet; i.p.: intraperitoneal; LC-MS-MS: liquid chromatography–tandem mass spectrometry; MTT: 3-(4,5-dimethylthiazol-2-yl)-2,5-diphenyltetrazoliumbromide; NR: neutral red; P-gp: P-glycoprotein; PCR: polymerase chain reaction.
